# Bio-Based Packaging: Materials, Modifications, Industrial Applications and Sustainability

**DOI:** 10.3390/polym12071558

**Published:** 2020-07-14

**Authors:** Corina L. Reichert, Elodie Bugnicourt, Maria-Beatrice Coltelli, Patrizia Cinelli, Andrea Lazzeri, Ilaria Canesi, Francesca Braca, Belén Monje Martínez, Rafael Alonso, Lodovico Agostinis, Steven Verstichel, Lasse Six, Steven De Mets, Elena Cantos Gómez, Constance Ißbrücker, Ruben Geerinck, David F. Nettleton, Inmaculada Campos, Erik Sauter, Pascal Pieczyk, Markus Schmid

**Affiliations:** 1 Sustainable Packaging Institute SPI, Faculty of Life Sciences, Albstadt-Sigmaringen University, Anton-Günther-Str., 51, 72488 Sigmaringen, Germany; reichert@hs-albsig.de (C.L.R.); sautere@hs-albsig.de (E.S.); pieczypa@hs-albsig.de (P.P.); 2 IRIS Technology Solutions S.L., Parc Mediterrani de la Tecnologia, Avda, Carl Friedrich Gauss 11, 08001 Barcelona, Spain; ebugnicourt@iris.cat (E.B.); david.nettleton@iris.cat (D.F.N.); icampos@iris.cat (I.C.); 3 c/o Department of Civil and Industrial Engineering of University of Pisa, Inter University Consortium of Materials Science and Technology (INSTM), Largo L. Lazzarino 1, 56122 Pisa, Italy; mb.coltelli@ing.unipi.it (M.-B.C.); patrizia.cinelli@unipi.it (P.C.); andrea.lazzeri@unipi.it (A.L.); 4 Planet Bioplastics s.r.l., via San Giovanni Bosco 23, 56127 Pisa, Italy; ilariacanesi@planetbioplastics.com; 5 Laboratori ARCHA Srl, via di Tegulaia 10/A, 56121 Pisa, Italy; francesca.braca@archa.it; 6 Aimplas, Plastic Technologic Center, Valencia Parc Tecnologic, C/Gustave Eiffel, 4, 46001 Valencia, Spain; bmonje@aimplas.es (B.M.M.); ralonso@aimplas.es (R.A.); lagostinis@aimplas.es (L.A.); 7 OWS, Dok-Noord 5, 9000 Ghent, Belgium; steven.verstichel@ows.be (S.V.); Lasse.Six@ows.be (L.S.); steven.de.mets@ows.be (S.D.M.); 8 European Bioplastics, Marienstrasse 19/20, 10117 Berlin, Germany; cantos@european-bioplastics.org (E.C.G.); issbruecker@european-bioplastics.org (C.I.); 9 Centexbel, Technologiepark 70, 9052 Zwijnaarde, Belgium; rg@centexbel.be

**Keywords:** bioplastic, packaging, textile, polylactic acid, coatings, biodegradation, upscaling, end of life

## Abstract

Environmental impacts and consumer concerns have necessitated the study of bio-based materials as alternatives to petrochemicals for packaging applications. The purpose of this review is to summarize synthetic and non-synthetic materials feasible for packaging and textile applications, routes of upscaling, (industrial) applications, evaluation of sustainability, and end-of-life options. The outlined bio-based materials include polylactic acid, polyethylene furanoate, polybutylene succinate, and non-synthetically produced polymers such as polyhydrodyalkanoate, cellulose, starch, proteins, lipids, and waxes. Further emphasis is placed on modification techniques (coating and surface modification), biocomposites, multilayers, and additives used to adjust properties especially for barriers to gas and moisture and to tune their biodegradability. Overall, this review provides a holistic view of bio-based packaging material including processing, and an evaluation of the sustainability of and options for recycling. Thus, this review contributes to increasing the knowledge of available sustainable bio-based packaging material and enhancing the transfer of scientific results into applications.

## 1. Introduction

Plastic is most often the material of choice for the packaging of food, cosmetics, or pharmaceutical products based on its low cost, light weight, and the excellent protection provided to the packaged product [1]. In general, petrochemical plastics provide excellent functionality for use as packaging materials, including mechanical and barrier properties as well as low production costs [2]. Applications of bio-based materials in the sustainable packaging industry have shown strong growth due to recent trends in the consumer market moving toward greener packaging and reduction of waste. In general, bio-based plastics are defined as human-made or -processed organic macromolecules derived from biological resources [3]. Amongst the plastic produced annually, bio-based plastics represent only 1% in Europe [4]. Notably, the demand for bio-based materials for packaging is expected to grow up to 9.45 million tons by 2023 [5]. The issue of sustainability is crucial, encouraging politics, academia, and industry to develop sustainable and circular alternatives for preserving resources for future generations, focusing on biodegradable and bio-renewable materials.

Several methods are available for enhancing the sustainability of packaging material. One is to reduce the raw material needed by thinning the packaging film or designing the packaging by minimizing the amount of material used for the packaging production. Both lead to lighter packaging and thus to reductions in material resources, costs, and energy [6]. Using this approach reduces the amount of plastics used, which are most often sourced from fossil raw materials. Another method involves using post-consumer recycled material or reusable packaging, thus enhancing the recycling of the packaging material [6,7]. Further enhancement in sustainable packaging performance was reached using multilayer packaging. Multilayers combine lower amounts of packaging material and improved barrier properties due to the use of several layers able to provide different barrier properties to protect the packaged product.

However, all the mentioned methods are limited and unsuitable for producing a packaging material that is completely sustainable and fulfills the requirements of a circular (bio)economy. For instance, multilayer packaging material often has poor recyclability, necessitating incineration or being sent to landfill [8]. Thus, the demand is strong for bio-based material from renewable sources that are organically, chemically, or mechanically recyclable.

Innovative packaging materials composed of blends or pure bio-based materials are expected to improve the sustainability of these products. Using renewable resources for the development of bio-based packaging material produces a smaller carbon footprint, reduces environmental impact [6], increases acceptance by consumers, maintains barrier properties and shelf-life of the packaged good, and allows for a sustainable end of life [9]. In general, two different routes can be stressed for increasing the ecological performance of packaging materials: one route describes the need for new material sources, which is biomass instead of petrochemical sources, and the second route describes alternative end-of-life scenarios. An end-of-life scenario can include either incineration, landfilling, recycling, or composting. A review article evaluating the sustainability performance of bio-based plastics concluded that bio-based plastics have the potential to save up to 315 million tons of CO_2_ equivalents annually [10]. However, to date, several barriers exist to their implementation, such as the limited sorting or recyclability of bio-based materials or land-use needed for bio-based materials competing with food production etc., all of which influence the sustainability assessment of bio-based materials [2].

Thus, innovative packaging concepts include novel materials in mono- or multilayers while maintaining biodegradability. Novel materials to be used for bio-based packaging should be bio-based, approved for food contact, or biocompatible and biodegradable for medical applications. Bio-based sustainable packaging material should ideally be derived from renewable resources or side streams from processing of agricultural or food products, which are receiving considerable attention in industry and academia and are not competing with primary food production. Based on the sources used for producing packaging material or textiles, we can differentiate between first-, second-, and third-generation feedstocks based on the classification of biofuels. First-generation feedstock implies biomass that is generally edible such as sugarcane, whey, or maize. Second-generation feedstock, including mostly lignocellulosic-based feedstocks, includes biomass that is mostly non-edible such as residues or side stream products from agriculture, forest, and animal industry, as well as municipal wastes. Third-generation feedstock includes biomass from algae [11].

**Figure 1:**
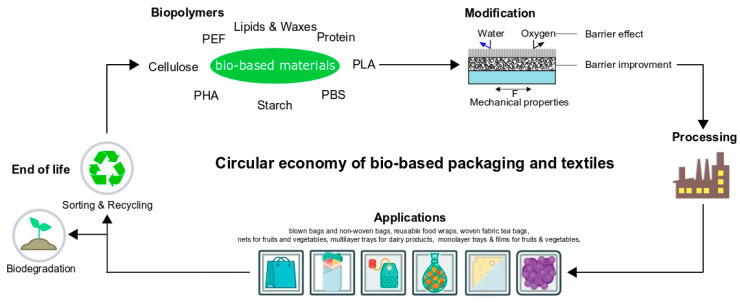
Schematic illustration of an ideal cycle of bio-based material to be used for packaging application (PLA: polylactic acid, PEF: polyethylene furanoate, PBS: polybutylene succinate, PHA: polyhydrodyalkanoate).

In most cases, the decrease in price, decrease in competition with food, but increase in complexity to use the source are associated with increases in the generation of feedstock. An ideal bio-based packaging material cycle is schematically illustrated in Figure 1.

For instance, whey protein, a by-product of cheese processing, has been extensively studied as packaging material [12,13,14,15,16,17]. The detrimental barrier properties of whey protein-based films can be improved and tailored by physical modification of the proteins. Physical modifications include heat treatment [18], ultrasonic treatment [19], or irradiation [20] to create protein films and coatings. Developments have occurred in chemical modification of proteins especially by chemical grafting using fatty acids to improve the water vapor barrier properties [21]. Besides whey proteins, proteins from soy and peas [22] as well as polysaccharides such as cellulose and starch [23] have been analyzed regarding their packaging film properties.

Among the proteins and polysaccharides, polyesters are some of the most extensively studied polymers derived from biomass. Polyhydroxyalkanoate and newly developed materials, namely polyethylene furanoate, have been considered for various packaging applications despite being non-biodegradable [24]. To use bio-based and biodegradable polyesters for large-scale production, developments in their processing and upscaling were outlined in another review [25]. Polylactic acid (PLA) is one of the most studied synthetic polymers already applicable for various industrial packaging applications. The main disadvantage of PLA is its slow biodegradation in mild conditions, which is strongly dependent on hydrolysis, thus requiring industrial composting conditions [26].

Many textile applications (e.g., teabags and fruit/vegetable nets) are based on PLA as PLA has one of the highest heat resistances and mechanical strengths of all bio-based polymers, except for the drop-in polymers [27].

The aim of this review was to illustrate the state-of-the-art advances in bio-based packaging and packaging material, as well as modification strategies and application scenarios. To fully assess the sustainability of bio-based packaging material, we outline and discuss the evaluation of different end of life options including home and industrial composting in this review. We outline the advances in the development of bio-based packaging material for specific applications.

## 2. Bio-Based Materials for Packaging Applications

Many efforts have been directed toward commercializing novel biopolymers with improved properties and new functionalities for packaging films and coatings as well as textile applications. Biopolymers can be produced using different methods: either directly from natural substances that include polysaccharides and proteins, or by polymerization of monomers derived from biomass, such as PLA from lactic acid. Other biopolymers can be produced by microorganisms such as polyhydroxyalkanoate (PHA) [28].

In the following, the most promising biopolymers for packaging applications are outlined and modification techniques are presented, if appropriate. In addition, we summarized key parameters of the bio-based materials for packaging applications in Table 1.

### 2.1. Synthetically Produced Bio-Polyesters

In contrast to natural biopolymers that are produced intra- or extra-cellularly in living organisms (Section 2.2), synthetic biopolymers can be built via synthesis reactions.

#### 2.1.1. Polylactic Acid (PLA)

PLA-based polymers are one of the most extensively studied synthetic polymers from biomass such as from corn stover, sugarcane bagasse, or rice hulls, which are the most frequently used feedstock for PLA production [30,54]. PLA-based polymers are used for a wide range of applications including disposable household items, food packaging, agricultural films, drug delivery systems, and implantable biomedical devices [55]. Polylactic acid, as an aliphatic polyester, can be synthesized by lactic acid (LA) or by lactide. LA, or 2-hydroxypropanoic acid, provides hydroxyl and carboxylic acid functionalization that can lead to intermolecular esterification reactions. Self-esterification of LA at 120–135 °C leads to formation of the cyclic dimer lactide, or 6-dimethyl-1,4-dioxane-2,5-dione, which is insoluble in water [32].

PLA is produced with a tunable stereochemical structure that may be easily modified by controlling the ratio of its L- or D-isomers during the polymerization, which affects the melting temperature and the ability to crystallize. In industrial packaging applications, the use of PLA with a randomly distributed D units’ content of 2–6 wt% is common. With this formulation, the crystallinity of PLA is low, favoring the production of amorphous transparent films. PLA might be synthesized by polycondensation or ring-opening polymerization depending on the monomer used [32,33]. Lactic acid is directly synthesized by polycondensation in bulk by distillation of condensed water during continuous increase of vacuum and temperature. If necessary, a catalyst is added, while vacuum and temperature are progressively increased. A polymer of low molecular weight and uncontrolled stereoregularity is produced, which is the main disadvantage of direct polycondensation that restricts its use [56].

For commercial production of PLA, the ring-opening polymerization technique is usually selected. This method achieves lactide polymerization via a catalyzed coordination–insertion mechanism [57]. This is the most preferred route for producing high-molecular-weight PLA on an industrial scale [58,59]. Ring-opening polymerization has been successfully achieved using melt, solution, bulk, and suspension techniques [32].

NatureWorks LLC (Minnetonka, MN, USA) developed a continuous process to produce PLA from corn-derived dextrose on an industrial scale. The resulting commercial PLA (Ingeo^®^) was the first synthetic polymer to be produced from renewable resources on large industrial scale [56,60]. The application of the reactive extrusion of PLA is a new specialty in ring-opening polymerization reactions that has recently emerged in small-scale investigations as a tool, having the potential to replace the chemical batch reactor. The advantage of extrusion polymerization is the solvent-free and low-cost continuous process [61].

For textile applications, high crystallinity is needed to prepare fiber structures [32]. Fibers composed of PLA show the most potential to be used as the raw material for textiles due to their similarity to other thermoplastics [62]. For example, the mechanical properties of polyethylene terephthalate (PET) fibers are like fibers composed of PLA. PLA fibers have yarns with low moisture absorption and fast drying properties [63].

The initial degradation step of PLA occurs preferentially in amorphous regions, where the ester groups are hydrolytically degraded in the presence of water [64]. The carboxylic end groups act catalytically to affect the hydrolytic degradation of PLA in a self-catalyzed and self-maintaining process [65,66,67]. PLA becomes water-soluble when the molecular weight is below 20,000 g/mol and can then be uptaken by microorganisms, undergoing the metabolic process and converting it into carbon dioxide, water, and biomass [68,69,70].

The PLA composting process proceeds efficiently under adequate conditions due to the combined effects of hydrolysis and microbial activity [71]. A patent includes a protease variant that is especially suitable for PLA degradation [72]. The elevated temperature encountered during industrial composting is important and may accelerate the PLA hydrolysis process [73].

To date, large amounts of research are ongoing about the copolymerization of lactide molecules to improve the biodegradation of PLA. For instance, glycolic acid is the most studied co-monomer used with lactic acid [34,74,75]. Block co-polymerization of PLA with poly(ethylene glycol) (PEG) is another common technique used to enhance the biodegradability of polylactide [34,75,76,77]. A study revealed a solvent-free synthesis of a PLA–PEG block copolymer with enhanced biodegradability [78].

Efforts have been made to synthesize PLA from second- and third-generation resources, resulting in the development of different polyester-anhydrides [79]. Most of these are bifunctional dicarboxylic acids, such as malic acid, succinic acid, or itaconic acid, that are suitable candidates for copolymerization with lactide to obtain biodegradable copolymers. Dicarboxylic acids, such as malic acid or succinic acid, are inexpensive and non-toxic chemicals that are naturally present in vegetables and fruits [80]. Currently, 80% of global malic acid is produced synthetically and the remaining is produced from renewable sources, amongst others, from fruit waste. Malic acid is used to produce bio-based polyesters, which find application in pharmaceuticals used to treat cancer cells. Poly(malic acid-co-L-lactide) acts as a superb degradation accelerator for PLA in physiological conditions due to the superior ability of poly(malic acid) compared with PLA to biodegrade [81,82].

An innovative study of the synthesis of PLA-functionalized polymers starting from a derivative of malic acid O-carboxyanhydrate monomers was reported, presenting a new alternative for the preparation of PLA-based materials [83]. This synthetic pathway is of interest because it allows keeping the hydroxyl group of the malic acid free, which would otherwise be part of the ester bond of the main polymer chain, leaving a carboxylic group free instead, which is normally observed in copolymers with PLA [83,84,85]. Poly(malic acid-co-L-lactide) acts as a strong degradation accelerator for PLA under physiological conditions [85].

The industrial-scale production of succinic acid is reported to be viable by fermentation with bacteria, fungi, or yeast. Renewable raw materials, such as industrial waste, and by-product streams may be used as resources for succinic acid production [86]. Even if succinic acid is more commonly used in PLA blends [87], studies of their direct copolymerization have been published. For example, researchers investigated the biodegradation behavior of poly(lactic acid-co-ethylene-co-succinic acid) copolymers. The lack of methyl groups in the succinic molecule compared to lactic acid was reported to favor the biodegradation and the decomposition behavior of its derivatives, such as poly(butylene succinate), which has been well documented [88].

Finally, itaconic acid, or methylene succinic acid, is a diacid produced on an industrial scale via bio-based fermentation with *Aspergillus terreus*. Itaconic acid has been intensively studied as an alternative co-monomer for acrylic acid and methacrylic acid [89]. For example, Sood et al. (2017) [90] and Gupta et al. (2018) [91] reported the synthesis of a biodegradable hydrogel of poly-(lactic acid-co-itaconic acid) copolymerized using the microwave-assisted technique for drug delivery purposes [90,91].

In PLA and derived blends, selecting the appropriate plasticizer type and amount allows the modulation of the biodegradability [43]. The use of LA oligomers as plasticizers in PLA enables the production of valuable mechanical properties and biodegradability. Even the effect of nucleating agents, such as poly-D-lactide [92], talc, calcium carbonate, or even montmorillonites [93,94], etc., are being studied to adjust the crystallization and thus to modulate the biodegradation properties of PLA. The degree of crystallinity of PLA films highly influences its water vapor transmission rate (WVTR): amorphous PLA films (calculated to a film thickness of 100 μm; 0% crystallinity) provides twice the WVTR (43 g/(m^2^⋅day) at 25 °C) compared to high crystalline PLA films (21 g/(m^2^⋅day); 25 °C; 66% crystallinity) [95].

Many studies focused on the enhancement of PLA to address PLA’s disadvantages as packaging material such as its brittleness and poor gas and UV barrier properties For instance, PLA was blended and compounded for instance with naturally obtained fillers and additives to modulate PLA’s properties [96]. A detailed description of the state of research about PLA films is out of the scope of the present article. Current research about PLA and its potential to replace petrochemical plastics are outlined elsewhere [54,97] and current industrial applications of PLA in packaging material and textiles are outlined in Section 4.

#### 2.1.2. Polyethylene Furanoate (PEF)

Polyethylene furanoate (PEF) is a 100% bio-based polyester that is polymerized from 2,5-furandicarboxylic acid (FDCA) and monoethylene glycol (MEG). FDCA is a monomer derived from sugars using biofermentation, which can replace oil-based terephthalic acid (TA) in polyethylene terephthalate PET to produce PEF [35,36].

To produce PEF, first-generation bio-feedstock, such as corn- or wheat-based sugars, or second-generation feedstock, such as waste, wood, wheat-straw, corn stover, or bagasse, can be considered when they become economically viable [35]. PEF has barrier and thermal resistance properties comparable to PET [98]. PEF provides twice as high a water vapor barrier than PET [36]. The glass transition temperature is 86 °C for PEF and 74°C for PET [99]. The melting temperature of PEF at 235 °C is lower than for PET at 365 °C [99]. In a scientific opinion, the European Food Safety Authority (EFSA) assesses the safety of a substance before it is authorized for use in food contact. In this assessment, the EFSA panel verified the safety of PEF with the following wording “the use of the substance furan-2,5-dicarboxylic acid as a monomer in the production of PEF polymer does not raise a safety concern for the consumer when the migration of the substance itself does not exceed 5 mg/kg food” [100].

#### 2.1.3. Polybutylene Succinate (PBS)

Polybutylene succinate (PBS) is a polyester formed by polycondensation. PBS has high crystallinity and good thermal properties, with mechanical properties similar to those of polypropylene (PP) [37]. Whereas PBS was produced for a long time with the help of petrochemical materials, the synthesis of bio-succinic acid by yeasts or bacteria such as *Anaerobiospirillum succiniciproducens* has been patented [38]. PBS can also be used as an additive for plasticizing other bio-polymers such as PLA [39].

### 2.2. Non-Synthetically Produced Polymers

Non-synthetic or natural polymers, which are produced by living organisms, mainly include polyesters, proteins, and polysaccharides.

#### 2.2.1. Polyhydroxyalkanoate (PHA)

PHAs are microbial polyesters that can be synthesized by a wide range of microorganisms under conditions of nutrient stress through fermentation of sugars, with thermoplastic properties similar to those of conventional plastics, using a variety of microorganisms [9]. PHAs are a family of biopolymers, among which some types are already available on the commercial scale such as poly(3-hydrocybutyrate) and polyhydroxybutyrate-co-valerate. An overview of PHAs that are produced on industrial scale were summarized elsewhere [42]. PHAs are 100% bio-based and biodegradable. The properties of these polyesters can be tuned over a wide range by varying their chemical compositions. The microbial production of PHA occurs in several steps, where the properties can be adjusted by adjusting the substrate and the fermentation conditions [101]. Advances in molecular and genetic engineering led to the selection of several bacterial strains able to produce PHA in high amounts, over 90% of total biomass, such as *Ralstonia Eutropha,* or *Alcaligenes Latus* [43]. So far, the application of PHAs is limited due to their relatively high costs (EUR 2.2–5.0 kg^−1^ [42]). Thus, PHAs are mainly used for high-value applications such as pharmaceutical or medical products. The increasing industrial production of PHAs in emerging countries, mainly in China, will result in a reduction in the cost of the PHAs soon. The addition of low-cost natural and inorganic fillers can allow the production of cost-sustainable PHA-based composites that can be applied in single-use products where PHA biodegradability in compost, soil, and marine water represents a main advantage in, for example, packaging and agriculture [43].

PHAs can be used raw, blended, or as an additive to modify other polymers such as PLA, PBS, polycaprolactone, etc. To produce bioplastics made from PHAs, the biopolymer has to be extracted from the bacterial cell material, cleaned, and compounded [9]. PHAs can be produced with either a high or low molecular weight depending on the application. For thermoforming or blow molding, PHAs with a high molecular weight are preferred based on their higher mechanical strength compared to low-molecular-weight PHAs. In comparison, PHAs with low molecular weight were shown to be preferable for injection molding applications [102].

The chemical composition of PHAs can be modified in terms of the combinations of monomer subunits depending on the nutrients provided in the fermentation step. For industrial production, mostly pure cultures and refined substrates are used, leading to expensive PHA production. Thus, some studies investigating mixed microbial cultures and no-cost feedstock as the substrate, such as wastewater, to produce PHA were reviewed [42].

PHAs can be produced either as homopolymers or copolymers. Homopolymers are composed of one type of (PHA) monomer, for instance, pure poly(3-hydroxybutyrate) (P3HB) or poly(4-hydroxybutyrate (P4HB). Copolymers, conversely, are built of two or more different PHA monomers. PHAs that are produced on an industrial scale are mostly composed of P3HB and one other PHA as the co-monomer [25].

The properties of the hydrophobic PHA polymer include heat and ultraviolet light resistance, and some of the industrially produced PHAs are approved for food contact. PHAs are durable for high-speed processing and can withstand high temperatures during storage. Binding with other materials (e.g., paper) is possible [103,104]. Some degree of market penetration has been achieved in the production of packaging, coatings, and hygiene products composed of PHA [25]. The blending starch and PHA was reported to be promising for producing active cosmetic films compatible with skin [105]. PHA blends with PLA were found to provide water barrier properties close, but still slightly inferior, to those of commodities [106].

PHAs can be designed for different end-of-life solutions: either composted in an industrial composting plant or biogas installation, or on a home composting heap. Some PHAs can biodegrade in different environments (e.g., soil or marine environments) [9]. A review summarized information about the biodegradation of PHA in marine environments and, based on a meta-study, concluded that the speed of marine biodegradation of PHA is between 0.04 and 0.09 mg⋅day^−1^⋅cm^−2^ (*p* = 0.05) [44].

#### 2.2.2. Cellulose and Derivatives

Cellulose, a polysaccharide, is one of the most used biopolymers for alternative natural packaging material. Cellulose is composed of β-D-glucose subunits and its polymers are obtained from plant material. In its native form, cellulose has a very low water solubility and thus is a rather unsuitable packaging material. Solubility is needed to process a substance and reconstitute it into a film or fiber to be used as material for packaging or textile materials [47]. However, cellulose can be modified by plasticizing, surface modification, or coating and blending to become water soluble. Thus, cellulose modified with the addition of plasticizers provides a raw material for packaging film formation [23]. Nanocellulose fibers can be developed from cellulose by acid hydrolysis or mechanical grinding [45]. Nanocellulose fibers provide high mechanical stability and optical transparency. From the ~15–20-nm-thick fibers, films and composites can be produced [46]. The fibers from nanocellulose can be used in composites, preventing the need for the use of inorganic fillers [81,107]. The incorporation of nanofibers into the polymer matrix can improve the poor moisture barrier of cellulose without affecting biodegradability [46].

Cellulose can be modified into regenerated cellulose fibers also called rayon. This type of fiber was the first human-made fiber developed in the 1850s. Due to competition of other human-made fibers, the market share of rayon fiber has decreased to about 3 million metric tons per year, which is about 5% of the global human-made fiber production. Four fiber types are produced from cellulose and its derivatives: viscose, lyocell, cupro, and acetate. Viscose is characterized by good drapability, softness, and moisture absorption. However, viscose is easily stretchable with poor recovery, and has low abrasion and wrinkle resistances. Lyocell was developed due to environmental concerns and has better drapability and feel, and higher tensile strength [108]. Cellulose acetate fiber is a cellulose derivative where some of the alcohols are replaced with acetate groups. If two groups are replaced, a diacetate fiber is produced; when replacing three groups, a triacetate fiber is obtained. The difference between the fibers is that triacetate has a lower wet break elongation, lower moisture regains, and better wrinkle resistance. Both fibers have good dimensional stability [108].

#### 2.2.3. Starch

Starch is a biopolymer produced by the photosynthesis of plants. Starch is present in granules within plant cells that have both amorphous and crystalline regions. Starch is composed two homopolymers: amylase and amylopectin. Amylose is a linearly structured polymer of D-glucose monomers. Amylopectin is an α-D-(1-4)-glucan, which has α-D (1–6) linkages at the branch point. One of the most used commercial starches is obtained from potatoes; other common sources are corn, wheat, rice, and tapioca. Depending on the plant source, starch is composed of different amylose-to-amylopectin ratios ranging from about 10% to 20% amylose and 80% to 90% amylopectin. Starch granules consist of crystalline and amorphous regions, with the amorphous regions mainly containing amylose linear chains and amylopectin branching points. The crystalline regions are mainly composed of long amylopectin side chains. Starch can be easily dispersed in cold water and, upon heating, a starch and water mixture; the starch forms a colloidal gel network during cooling. The glass transition temperature of pure, dry starch is above the decomposition point, so starch does not soften and flow. However, starch can be plasticized (de-structured) by molecules that interact via hydrogen bonding. Even low concentrations of these molecules (15–30 wt%), such as water, glycerol, and sorbitol that interact with the hydroxyl group of the starch, are needed to improve the mechanical strength and the barrier against water.

This thermoplastic starch (TPS) flows at high temperature and pressure; thus, TPS can be extruded to produce both foams and solid molded products. One of the disadvantages of TPS is its brittleness caused by ageing during storage. This brittleness increases with time due to retrogradation.

Starch is biodegradable in a wide variety of environments such as soil and water/seawater [50]. The biodegradability mechanism is influenced by environmental parameters such as the microbiome and fungi present, as well as external factors such as temperature, light, oxygen, water, pressure, ozone, etc. [51]. In general, starches degrade into glucose by microorganism or enzymes, and then are metabolized into carbon dioxide and water.

A study used potato starch as the raw material to develop new packaging materials. The BIOPLAST products, which are manufactured in conventional production plants developed by BIOTEC GmbH & Co. KG (Emmerich am Rhein, Germany) can be produced with different purities of bio-based carbon contents, which determine if the potato starch packaging material can be composted industrially or at home [109].

#### 2.2.4. Proteins

**Figure 2:**
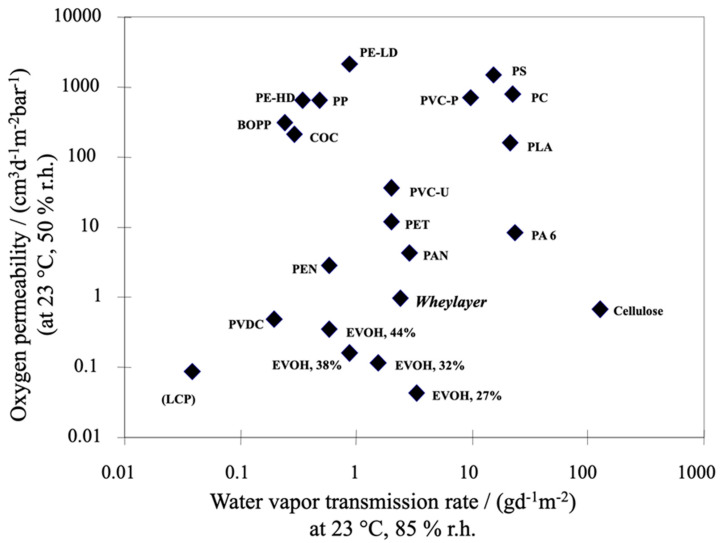
Permeability values of typical plastics, bioplastics, and Wheylayer^®^ normalized to 100 µm thickness [15,17].

Proteins were proven to be an effective alternative coating and film for packaging applications [110]. In former European projects named WHEYLAYER [111] and THERMOWHEY [112], oxygen barrier coatings based on whey protein were developed. Whey continues to be a widely available and underused by-product from cheese-making, containing around 13% protein in dry matter. The whey-protein-based coatings are formulated using whey protein isolate (WPI) as well as bio-based plasticizers and/or reactive additives, such as urea, to enhance thermoformability [113,114]. The whey formulation is subsequently coated on a plastic substrate and dried. In this process step, the protein is denatured to form a crosslinked network. The coated film is laminated into the final structure and, eventually, additional conversion processes, such as thermoforming, sealing, etc., can be carried out to produce the packaging items. Resulting films were reported to have excellent mechanical as well as gas and UV barrier properties, beyond those of most other biopolymers (Figure 2). Whey coatings, among which those upscaled and trademarked Wheylayer^®^, are thus capable of replacing synthetic oxygen-barrier layers such as EVOH in multilayer packaging. The whey coating is both bio-based and biodegradable [12,14,15,17].

On PLA, based on preliminary tests, a 100-fold barrier improvement factor was obtained using coating. The use of nitrogen-rich protein coatings on biodegradable based films does not compromise, but rather improves, the biodegradability of PLA [13].

Like whey protein, soy protein and pea protein isolate also consist of many globulins; however, they have a different amino acid composition. This leads to the protein film varying in structure based on the source of protein, providing different oxygen permeabilities. Films that are composed of plant protein isolates have excellent oxygen barrier properties due to their polar nature and crosslinked polymer network; however, the water vapor barrier is low [16,22]. Chang et al. showed that whey or pea protein isolate film coatings have lower oxygen permeability compared to multilayers containing nylon as the oxygen barrier: The multilayer PET/WPI/cast polypropylene package has 90 times lower oxygen permeability compared to the PET/nylon/cast polypropylene control multilayer. The PET/pea protein isolate/nylon/cast polypropylene CPP multilayer showed nearly 1000 times better oxygen permeability [22].

Although films and coatings composed of proteins have already shown considerable potential in many respects, additional modifications, such as physical, chemical, and enzymatic modifications, are useful for tuning mechanical and barrier properties.

The passage of oxygen through a layer with different partial pressures can be tuned, for example, by treatments using ultrasound [16,115,116], heat [117,118,119], and others [14]. For instance, a low frequency ultrasonic treatment of soy proteins resulted in a higher protein solubility and a modified surface [120]. Weng and Zheng observed higher vapor and oxygen barriers of films containing modified soy proteins [121]. An approach to improve the water vapor barrier of bio-based films and coatings involves modifying protein films by fatty acids. The fatty acid grafting can be applied on protein-based films and coatings using the transfer method, which was previously described for surface grafting of PVOH-based films [14]. Another approach to improve barrier properties is the application of enzymes such as transglutaminase to form more crosslinked protein films as previously shown for whey proteins [122,123], as previously cited [16]. However, other studies revealed that increasing the degree of hydrolysis of whey protein using peptidase decreased the mechanical properties but barely influenced the barrier properties [101,102]. The water vapor transmission rate of whey protein films was improved by coating with hydrophobic material such as beeswax and acetylated monoglycerides [124,125], as previously cited [16].

Studies with montmorillonite nanoclay, a bio-nanocomposite, showed that the mechanical properties of protein films may be improved by the addition of a montmorillonite nanoclay layer. Compared to untreated soy protein isolate films, water vapor permeability, tensile strength, and elongation at break can be improved [126,127,128,129]. Researchers found that the formulation of WPI films with the nanocomposite TiO_2_ had higher tensile strength but provided less of a moisture barrier [19,130].

Mechanical properties of protein films and coatings were found to be tunable by heat treatment [117,118,119]. For instance, heated whey protein films have higher tensile strength and increased rigidity but lower oxygen permeability [18,131,132].

Ultraviolet and γ-irradiation treatments also influence proteins’ structure and thus their film properties [133,134]. For instance, irradiation of protein films led to an increase in tensile strength without affecting the barrier properties of the tested soy and whey protein films [20].

#### 2.2.5. Lipids and Waxes

Lipids are hydrophobic substances such as fats, fatty acids, or waxes. Due to the non-polar regions of lipids, they provide an excellent barrier against moisture migration [135], as cited in Ashok et al. [136]. Most lipids used for films and coatings are composed of fatty acids with a carbon length between 14 and 18 [52]. The addition of further biopolymers, such as polysaccharides or proteins, as coatings to the lipids leads to better mechanical properties. Apart from the increased moisture permeability of blends compared with pure lipid coatings, blends offer higher barrier properties [137]. Waxes, such as paraffin wax from crude petroleum or beeswax from honeybees, prevent the passage of moisture or oxygen into food and lead to a smoother outer surface. Since the consumption of these waxes is harmless for humans, they are often used in edible films or coatings, which are used for fresh vegetables and fruit, amongst other products [53]. The mechanical properties were improved by using a smaller particle size of beeswax [131] as cited in Zink et al. [16]. The moisture barrier of candelilla wax was found to be better than that of conventionally used materials such as polyvinylidene chloride [138].

Above all, bio-based materials for packaging applications are currently being investigated as outlined above. Many materials are unsuitable for direct used as packaging material due to providing an insufficient barrier or insufficient mechanical properties. Thus, modifications, composites, or multilayer materials are needed, creating to the issue about the exact definition of biobased. There is no clear definition of the term biobased. According to European Bioplastics, “The term ‘biobased’ means that the material or product is (partly) derived from biomass.” [139]. However, the amount of biomass-derived material is not clearly defined. An ideal description of a bio-based sustainable material was given in the Introduction (Figure 1). Bio-based materials for packaging application often compete with primary food production, i.e., starch or sugar, creating to a demand for studies of packaging materials composed of second- and third-generation feedstocks.

Another term used to describe the environmentally-friendliness of materials is *sustainable*. According to the European Commission “Sustainable development means meeting the needs of the present whilst ensuring future generations can meet their own needs” [140]. An evaluation of sustainability is often lacking or is extremely complex and needs to be assessed for each individual packaging and the corresponding petrochemical counterpart. A more in-depth discussion on sustainability issues is provided in Section 5.1.

## 3. Materials Modification, Treatment, and Processing

### 3.1. Biocomposites, Fillers, and Additives

Biocomposites using natural fibers or fillers have been researched intensively over the last years, yielding valuable and applicable products due to their biodegradability, low cost, low relative density, high specific strength, and renewable nature, perfectly aligned with the circular economy approach. Several examples of biodegradable composites using different types of matrixes are reported in the literature, e.g., starch-based blends, PLA, and natural reinforcements. A wide variety of lignocellulosic fibers from agricultural and industrial crops, such as corn, wheat, bagasse, orange and apple peel, abaca, kenaf, hemp, flax, rice husk, pine, and jute, have been used in the production of composites in various industrial sectors, such as packaging, the automotive industry, and building and food by-products such as potato and legume pulp [141,142,143]. Natural fibers from plants are mainly composed of cellulose (50–70 wt%), hemicellulose (10–20 wt%), lignin (10–30 wt%), pectin, and small amounts of waxes [144].

The physical and mechanical properties of fibers vary depending on the plant source and thus may vary in density (approximately 0.8–1.5 g cm^−3^, elastic modulus (approximately 5–20 GPa), tensile strength (approximately 200–900 MPa), and elongation at break (approximately 1.5–20%) [145,146].

The use of fibers or fillers derived from food by-products is receiving attention due to increased production of starch and protein extracted from vegetal sources and proteins from legumes as integrators of vegetarian and vegan diets. The circular economy approach of valorizing by-products supports the valorization of natural fillers such as biochar from agricultural waste [147,148] for biocomposites. For instance, Poulose et al. studied the effect of a second-generation feedstock, date palm waste-derived biochar, as a filler material (5–15%) in polypropylene matrices. The results revealed that, as a filler, biochar changes some properties such as the tensile strength and reduces the ductility of biochar/polypropylene composites compared to neat polypropylene. The authors concluded that more research is needed to elucidate the filler–matrix interactions to produce superior composite properties [147]. Thus, research about bio-based fillers as bio-based packaging materials is needed because natural fillers considerably reduce the price of the final material since, generally, natural fillers are cheaper than bio-based polymeric matrices such as biopolyesters, in particular PLA, PBS, or PHA [149,150]. The addition of polysaccharidic fibers in biopolyesters, such as PLA-based blends, as reinforcing agent [149] is a well-known alternative for producing micro- or nano-biocomposites [151,152]. In general, the mechanical properties of a biocomposite result from the matrix and fiber properties as well as the adhesion between the fibers and the matrix [153,154]. The tensile strength highly depends on the adhesion between the fibers and the matrix, whereas the tensile modulus is highly influenced by the distribution and orientation of the fibers and their length-to-width ratio (aspect ratio). Thus, if proper compatibility is achieved and the fillers have a proper aspect ratio, the fillers can strengthen the material and increase the Young’s modulus. In addition to the tensile modulus, the fracture properties are strongly dependent on the aspect ratio. A critical fiber length determines the stiffness and strength of the composite. In contrast, fibers with a low aspect ratio and irregular shape behave more like fillers instead of a reinforcement. Natural fibers show a typical waviness that can slightly reduce the reinforcing action with respect to the well-known rigid and straight versions [155]. Composites promote biodegradability as the presence of fillers increases the polymer surface available for microbial attack, especially when the fillers are based on easily degradable natural components [156], such as cellulose or by-products with a high hemicellulose content [157]. The swelling and degradation of the fillers strongly promote the disintegration of composite material [158]. Studies on the biodegradation of PHBV-based composites with *Posidonia oceanica* as fillers in a simulated marine environment showed that the presence of *P. oceanica* fibers in the composites significantly accelerated the biodegradation of the polymeric matrix [159].

In biocomposites production, the amount of fillers loaded in a composite is limited by weak adhesion at the fiber–polymeric matrix interphase, poor mechanical properties, and difficulties in processing [160,161]. Therefore, the use of compatibilizers, such as maleic or itaconic anhydride, may allow the production of a higher load of fillers in the PLA polymeric matrix. As well as fillers pre-treatment, chemical, enzymatic, or coating approaches may improve the matrix–filler compatibility. In these approaches, the amount and properties of the coupling agent are crucial for the properties of the composites [156]. Even physical treatments such as stretching, or calendaring increase the interface of (natural) fibers but do not change the chemical composition of the fibers. Plasma or corona treatment can be used to enhance the compatibility of hydrophilic fibers and the hydrophobic matrix through formation of free radicals and surface cross-linkings. Coating fibers with hydrophobic compounds can be used to reduce the hydrophilicity of the lignocellulosic fibers. In addition, fibers and the matrix can be covalently connected via chemical treatments to reduce the fiber hydrophilicity or to increase the surface roughness. Treatments and coatings should be selected when targeting a green chemistry approach and bio-based coating of the natural fibers to preserve the biodegradability and sustainability of the bio-composites. For example, in biocomposites of PLA and potato pulp, the filler was not acting as a reinforcement due to the unfavorable aspect ratio of the potato pulp particles, which are short in length and irregularly shaped, and due to poor matrix–filler adhesion properties. Potato pulp coating with about 1 wt% of waxes improved the mechanical properties of the biocomposites and allowed for better matrix–filler adhesion; in particular, bio-based waxes based on bees or carnauba waxes resulted in bio-based compatibility between natural fibers and biopolyesters [162].

### 3.2. Processing of Bio-Based Materials for Packaging

In general, two main types of extrusion processing can be defined: single-screw extruders, which are applied for general polymer processing (blow molding, cast film extrusion, and injection molding), and twin-screw extruders, which are mainly used for compounding and polymer blending. Most of the polymer blends are produced using twin-screw extruders [163,164].

Following the increase in plastics production, the demand for larger extrusion capacity and better mixing performance drastically increased; therefore, twin-screw extruders have played a more important role in capacity increase than the single-screw extruders in the field of compounding and pelletizing since the early 1970s [164]. Some mixing elements in the screws can be applied to achieve different operational purposes with wide variety of materials. Studies on polymeric melt [165,166] stated that between co-rotating and counter-rotating twin-screw extruders, the former is more suitable for applications such as compounding, mixing, and chemical reactions due to the complexity of the flow in the intermeshing region, which provides good mixing and compounding characteristics. The morphology of polymer blends is known to influence their biodegradation rate [164]. One important aspect of biopolymer processing is controlling humidity: high humidity during polymer extrusion increases melt fluidity due to chain scission; thus, in industrial plants, drying sections are implemented. For extrusion, pellets of biopolyesters are usually heated in the presence of a dried air flow with a low dew point, while stirring the melting pellets to avoid undesired agglomeration [1].

**Figure 3:**
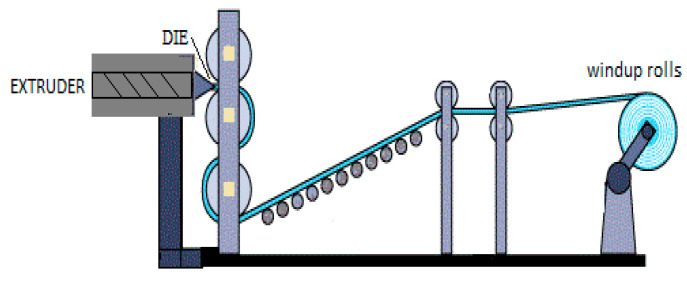
Schematic diagram of the flat die extrusion.

**Figure 4:**
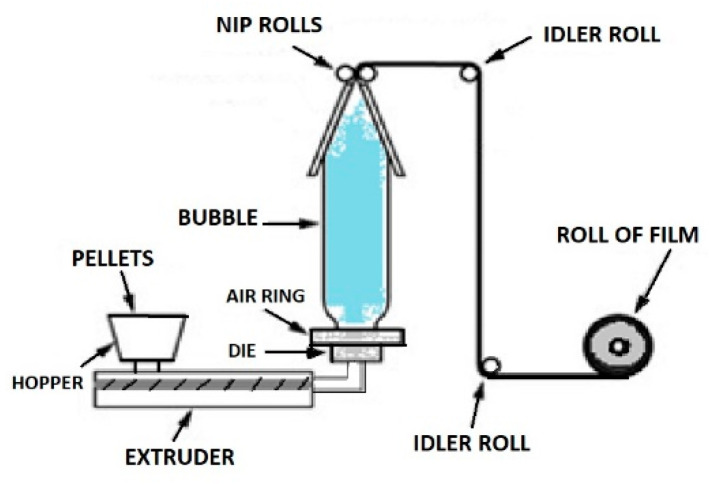
Schematic diagram of the blown film process [1].

In general, the production of flexible packaging is based on the preparation of plastic films using different converting processes, such as cast extrusion (Figure 3) and blown film (Figure 4). Flat die extrusion (Figure 3) allows the production of polymeric sheets and films (with a thickness ranging between 50 μm and 1 mm) and consists of the extrusion of the molten polymer through a linear die of rectangular geometry [1].

Blown film extrusion is used to produce pouches, industrial bags, or packaging films for shrink-wrapping because the produced film is tubular. The equipment consists of an extruder equipped with an annular die (Figure 4) [1].

The melt fluidity of PLA-based materials is generally too high to be processed with common blown film extrusion devices. Usually, chain extenders are used to improve the melt viscosity and strength to allow the production of PLA films by blown film extrusion. A patent reports the production of transparent PLA-based blends containing an epoxidized molecule acting as a plasticizer, compatibilizer, and fluidity regulator that provides good control of processability [167]. With respect to traditional polyolefins, the tearing strength of films composed of PLA-blends is lower. Recently, it was found to be possible to improve the tearing strength of PLA-blends, reaching values comparable to those of polyproylene using epoxidized molecules in combination with a nucleating agent [1,168].

**Figure 5:**
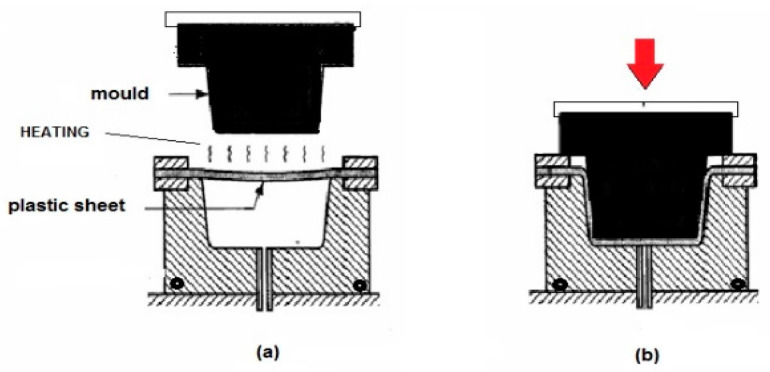
Thermoforming process [169].

For the production of rigid plastic containers, either injection molding or thermoforming (Figure 5) are used. In the injection molding process, polymer granules are heated, and the molten polymers are injected into a mold to produce pieces with a defined shape. Injection molding is used to produce, for example, caps, thick jars for cosmetics, cutlery, and coffee capsules [169]. A low viscosity at a high shear rate is required to enable the rapid and perfect filling of the mold. The melt flow index determination at the same temperature of injection molding can be a useful parameter in this process and, generally, thermoplastic polymers having a melt flow index above 10 g/min are suitable for injection molding. The temperature of the mold, and the holding pressure and time are the parameters that are important to control.

Biopolymers such as PLA and PHB can crystallize during the holding step, so the temperature of the mold and the holding time influence the crystalline morphology of the material. In turn, the amount and distribution of crystals in the material influence the material’s properties [169].

Contrary to injection molding, trays, plastic cups, blisters, and jars are produced by thermoforming (Figure 5). Polymer film, produced by flat die extrusion, with a thickness in the range of 50–300 μm is used for the thermoforming process. The material is usually heated by an infrared heater above its glass transition temperature but below its melting point to obtain a softened sheet. Then, a mold is inserted (or a vacuum is applied), which brings the softened sheet into the desired shape (Figure 5) [169].

### 3.3. Textile Production Techniques and Processing

Currently, only a small portion of bio-based materials are used for textile applications, the drop-In polymers. These polymers are identical to their oil-based counterparts. Examples include bio-PE, bio-PET, or bio-poly trimethylene terephthalate [170].

In addition to oil-based polymers, biopolymers show potential to produce textiles. Many of these polymers can be used to produce fibers and fragments that can completely or partly replace petroleum-based polymers due to their similar properties [28]. Examples of the most promising biopolymers for textile packaging applications are PHA, PBS, PET, cellulose, starch, and PLA. Fibers composed of PLA show the highest potential for use as raw material for textiles as they are similar to other conventionally used thermoplastics due to their smooth surface and their low moisture regain and mechanical properties [171].

Coatings are used on textiles for a number of aesthetic and functional purposes, and are generally not bio-based, e.g., polyurethane and polyamide [172]. For coatings, even PLA can be used for multiple applications as PLA has excellent UV stability and decent flame-retardant properties, which makes it useable for a broad range of interior and exterior applications. One of the main reasons why PLA coatings have not yet reached the market is their brittleness. For coatings, a molecular weight in the range of 10,000 g/mol is needed, whereas commercial PLAs have a molecular weight of about 100,000 g/mol. Thus, commercial PLA coatings need the addition of plasticizers. Plasticizers are not always compatible with PLA and issues may arise with the migration of the plasticizer. This causes increased brittleness over time as well as an unpleasant feeling coating. Migration of the plasticizer can be limited using highly compatible molecules and/or grafting them on PLA. Several initiatives have been undertaken to develop PLA coatings for textiles, but so far, PLA coatings are not commercially available [173].

**Figure 6:**
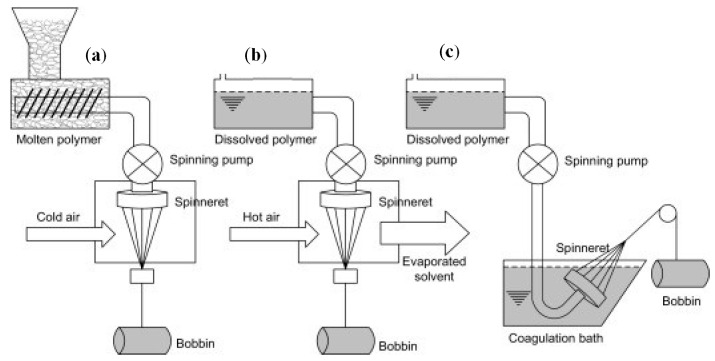
Spinning processes: (**a**) melt-spinning, (**b**) dry-spinning, and (**c**) wet-spinning (redrawn based on [172].

Different processes are available to produce yarns depending on the polymer type. An overview of the different spinning processes is schematically illustrated in Figure 6. The most used technique is melting spinning (Figure 6a). The thermoplastic polymer is melted, forming a thick, viscous liquid that is forced through the tiny holes of a spinneret to form continuous filaments. If a polymer cannot be melted, it can be dissolved to achieve a fluid state for extrusion (Figure 6b,c). If they cannot be directly dissolved or melted, they must be chemically treated to form soluble or thermoplastic derivatives.

In melt-spinning (Figure 6a), the polymer is cooled rapidly by air or water after the molten polymer is pushed through the holes of the spinneret. Polyamide, polyolefins, polyester, and many more polymers are produced in this manner [172]. Melt-spun fibers can be extruded from the spinneret in different cross-sectional shapes (round, trilobal, pentagonal, octagonal, and others). The extruded melt spinning technique is most frequently used for the production of fibers from biopolymers [172].

Through adapting and fine-tuning the extrusion process, the properties, such as the mechanical properties, of the yarn can be adjusted. The processing parameters that can be adapted are the following: extruder and die temperature, throughput, drawing rollers speed and temperature, die geometry, shear rate, melt and cold draw ratio, cooling rate of the molten polymer, spin finish application, etc. The success of the extrusion process can be determined via filament homogeneity, production speed, maximum drawing rate, mechanical properties, filament breakage, and observation of any anomalies.

In the EU-funded ECOBIONET project, the replacement of oil-based packaging nets with biodegradable and compostable nets was investigated. The formation from bio-polyesters and PLA was improved until the desired properties, such as tensile strength, were reached. In addition to the oriented nets, nets for shellfish products and nets in combination with plastic sheets were investigated. All of them can be produced by extrusion melt spinning [174].

## 4. Industrial Applications

Among the 322 million tons of plastic produced annually in 2015 worldwide, 58 million tons of plastic were produced in Europe with 40% of the plastic processed used for packaging with bio-based plastics accounting for only 0.5–1% of the EU annual plastic consumption [175]. To reduce the use petrochemically-based plastics, the food packaging industry is interested in the development of new bio-based materials to ensure that the food contained is safe and healthy and has optimal conservation. Big brand owners have introduced bioplastic packaging, including Danone (Actimel, Activia, Volvic), Coca-Cola (Plant Bottle), PepsiCo, Heinz, Tetra Pak, and L’Occitane.

Market research analysis forecasted an increase in biopolymers used for monofilms for flexible packaging consumed in Europe from a total 1743.9 million m^2^ in 2016 to 2427.1 million m^2^ in 2021. The increase in the use of biopolymer-based monofilms was forecasted to mainly occur in the category “Other food products incl. beverages” with an estimated increase of 161% within five years (2016 to 2021) [176]. The market drivers for growth are new packaging material developments with improved properties, greater availability, and lower price; the increasing awareness of environmental issues; and the adoption of new regulatory requirements. Using barrier materials supports one of the general trends in packaging, which is the decrease in packaging weight (thickness) to reduce the environmental impact due to resources efficiency and energy savings in transport. However, this trend poses challenges for material handling during recycling and further justifies the need for organic recycling as the preferred end of life. Sufficient barrier properties can also be ensured using bio-based materials such as the previously described coatings and modifications, the applications of which are summarized in the following sections according to their packaging format category.

Notably, in the following sections, we focus on pure bio-based packaging and not on partial bio-based materials.

### 4.1. Bio-Based Films and Trays

Progress has occurred in the development of films and trays composed of bio-based material for food packaging applications. A summary of suitable materials for bioplastic production was given elsewhere [177] as well as in Section 2. For instance, monolayer films composed of wheat gluten were studied [178]. Wheat gluten films were produced by traditional extrusion techniques, having mechanical properties that can be adjusted by pH with increased tensile strength properties at alkaline conditions (pH 9) [178]. Starch from potato or corn are in use for the development of film production. For instance, BIOME Bioplastics (Biome Bioplastics Limited, Southhampton, United Kingdom) offers bioplastics made of plant starches that can be processed at high temperature using methods such as injection molding, sheet extrusion, and thermoforming [179]. MATER-BI invented by Novamont S.p.A. (Novara, Italy) is a material composed of corn starch and oil that is compatible with the requirements for the production of films, bags, trays, cups, additives, foamed packaging material, extruded material, and injection molded material [180]. Another material that was intensively investigated as packaging material is PLA (see Section 2.1.1). A commercial PLA, trademarked Ingeo^®^ by NatureWorks LLC (Minnetonka, MN, USA), is used in market available products such as coffee capsules, diapers top-sheets or back-sheets, cups, yoghurt packaging, and electronics [60]. A multilayer film composed of PLA coated with a silicon oxide barrier was found to be suitable as cheese packaging material, preserving its quality for 65 days and thus providing an alternative packaging material to conventional plastic multilayer films [181]. Film packaging composed of PLA was found to be suitable for packing freshly harvested green peppers in modified atmosphere packaging [182].

Blends comprised of PLA and cellulose nanofibrils [183] and thermoplastic starch [184,185] have been studied as packaging material. Blends of cellulose nanofibrils, PLA, and casein as the compatibilizer were found to be a suitable as thermoformed packaging material based on increased tensile strength compared to bare PLA films [183,184]. Reis et al. studied the characteristics of trays composed of thermoplastic starch and PLA produced by flat extrusion, calendaring, and thermoprocessing with the trays covered with beeswax. The water vapor permeability markedly decreased through the hydrophobic beeswax coating of the tray. Thus, the trays, described as flexible and easy to handle, are suitable for use as packaging for fresh fruits and vegetables [184]. Another study revealed that blends of thermoplastic starch and PLA are suitable for sheet production when coated with cross-linked chitosan, providing a stronger water vapor barrier [185].

### 4.2. Bio-Based Pouches and Bags

Pouches made of bio-based material are currently scarce on the market. For instance, pouches were developed by BioBag International AG (Askim, Norway) as a multilayer composed of paper and layers of starch- and cellulose-based materials [186]. Another product was reported as the “first commercial bio-based spouted pouch” from erdbär Freche Freunde produced by GualapackGroup (Castellazzo, Italy). This eco-friendly pouch contains 100% organic fruit and vegetable pulps and addresses children. This “premade multilayer laminated pouch” is mainly composed of bio-PE [187].

A research article reported the successful production of bio-based pouches made of mango kernel starch using a casting technique as a suitable alternative to PE pouches to package red chili powder. The bio-based mango kernel starch pouches maintained the pungency and color of chili powder to a higher extent than when stored in PE pouches [188].

Plastic carrying bags represent contemporary modern culture and a product that is becoming regulated through bans or levies by many public authorities to reduce the amount of plastic bags used, as summarized previously [189]. Whereas the 2010 annual EU consumption was 10 billion pieces of multiuse bags, in recent years, shopping bags have evolved with the banning of single-use petrochemically-based non-biodegradable options in most EU countries. A Portuguese study revealed that the implementation of a tax for plastic bags led to a 74% reduction in the use of plastic bags. This study further reported that the tax did not have any environmental impacts such as marine littering [190].

Thus, bio-based materials for bags and pouches need to be more intensively investigated to produce a real effect on more sustainable and environmentally friendly bags. For example, bags for fruits and vegetables made of the bioplastic MATER-Bi are already on the market in Unicoop Firenze grocery stores in Italy [180].

### 4.3. Bio-Based Textiles and Nets

The use of biopolymers in textile applications is possible via extrusion and is estimated at 100 kt per year [4]. Conversely, thermoplastic starch is rarely used due to lacking the mechanical properties that are needed for most textile applications.

PLA fibers have several characteristics that are like many other thermoplastic fibers, such as controlled crimp, smooth surface, and low moisture regain. The physical properties and structure have been studied [171], and suitability as textile fiber has been acknowledged. Its mechanical properties are considered to be broadly similar to those of conventional PET [191] and, probably due to their lower melting and softening temperatures, comparisons to PP are also appropriated [192]. Some characteristics of a certain type of plastic can be a disadvantage in one application, and an advantage in another; for instance, the low water vapor barrier of the bio-based plastic PLA is a disadvantage for a water bottle but an advantage in packaging concepts for respiring products such as vegetables and fruits [193]. PLA filaments are available on the international market, but volumes are still limited. Examples of their producers include EcoDear PLA [194], Biofront (Teijin Fibers, Tokyo, Japan), PLA filament yarn (Polisilk S.A., Barcelona, Spain) [195], and Diolen (polyester PET high tenacity yarn; Swicofil HT PLA, Swicofil AG, Emmen, Switzerland) [196].

An innovative product made of PLA is teabags. These teabags are based on PLA non-wovens and can be industrially composted together with the tea waste. The material has the potential to replace paper-based teabags that commonly contain about 20–30% PP fiber to allow heat sealing. Despite ending up in home and industrial composting systems, current PP-containing tea bags are not compostable [193]. Pyramid tea bags composed of PLA yarn have been placed on the market by the Coats Group (RecLID Teabag, Coats Group, Uxbridge, United Kingdom) [197]. BioMesh, a non-woven material made of bio-resins, was developed by BIOME Bioplastics (Biome Bioplastics Limited, Southhampton, United Kingdom); however, information about constituents and characteristics have not been provided [179].

Nets are a widely used packaging format for fruits and vegetables. An EU research project named ECOBIONET aimed to produce biodegradable and compostable packaging nets made of biodegradable polyester materials including PLA with plasticizers and compatibilizers [174]. So far, some biodegradable nets are available on the market. For instance, BIO4PACK offers nets composed of starch- or cellulose-based materials that are reported to behave similarly to traditional plastic nets [198]. FKuR produces bio-based materials for nettings, for example, the bio-based and industrial compostable plastics made of blends based on PLA (Bio-Flex F 2110, Bio-Flex FX 1120, and Bio-Flex FX 1130) [199] and the bio-based and non-biodegradable Green HDPE SHE 150 made of high density polyethylene-green (HDPE) copolymer ethylene with 1-butene from BRASKEM [200]. Netco is also producing bio-based nets composed of PLA for vegetables (Bio-netting) [201].

All these applications are biodegradable under industrial conditions and more research is required to blend PLA with other polymers or the chemically change the PLA to make the end products home compostable.

## 5. Sustainability, Biodegradation, and Recycling

### 5.1. Sustainability

Numerous factors influence the degree of sustainability from sourcing until the end of life of the material, which are extensively discussed elsewhere [2]. To evaluate the sustainability of bio-based materials ranking, schemes and criteria need to be defined that are suitable for assessing its sustainability. The sustainability of a material depends on many factors from functional to aesthetic properties, environmental to economic and social benefits, impacts and costs, from production to end of life processes, and from local- to global-scale effects. Perfect sustainability is difficult, if not impossible, to achieve [2,202]. Ideally, products are based on renewable resources, are easy to reuse and/or recycle, and create added value without having adverse effects on our environment and population while maintaining a healthy profit for all supply chain actors. Bio-based, biodegradable plastics could meet some of these demands and can be produced from a broad range of feedstocks including maize, potatoes, sugarcane, cellulose [203], soy protein, chitosan, and agar [204], as well as from second- and third-generation feedstocks such as waste or wood, as outlined above (Section 2.1.2).

Several bio-based materials, including bioplastics, are considered competitive with conventional plastics in terms of global warming impacts [205]. In addition, bioplastics are competitive with synthetic counterparts with regards to costs, energy consumption, sustainability, and recycling procedure [203]. However, packaging that is sustainable implies high complexity based on widespread analysis and documentation evaluating the full life cycle of the sustainable bio-based material [203].

For PLA, a higher sustainability production system was not only achieved by production process optimizations, but also by switching to renewable energy sources to power the production system [206,207]. In general, debate is ongoing about assessing the “extent to which biodegradability and (home) compostability of plastic is beneficial in the context of the transition towards a circular economy” as outlined in a recent report by Eunomia. The issue regarding compostable packaging material lays in the risk of a possible increased littering of biodegradable plastics as well as undegraded compostable plastic residues. This report concluded that materials for product or packaging applications should be designed or chosen by emphasizing recyclability over compostability. The report stated that there are exceptions but, overall, more research is needed to elucidate the implications of compostable plastics in the waste infrastructure in Europe and how compostable materials can meet the requirements of a circular bioeconomy [208]. The production of bioplastics can be carbon neutral or even carbon negative due to the uptake of CO_2_ from the atmosphere during plant growth, followed by short- or long-term carbon storage in the product. For example, the Ingeo^®^ PLA biopolymer (NatureWorks LLC) is associated with an uptake of around 1.8 kg CO_2_/kg PLA [206,207]. Given this carbon uptake, the cradle-to-factory gate carbon footprint is around 2 kg CO_2_ eq. kg^−1^ PLA. With credits for using wind energy instead of fossil fuels, the carbon footprint of the production process is almost entirely neutralized. Technological improvements could further reduce resource needs, eventually reaching a negative cradle-to-factory gate carbon footprint in which more carbon is uptaken by the plants than emitted during the production processes [206].

The total impact of a product also depends on its use and end-of-life phases. The use phase strongly depends on specific product characteristics and regional factors. The impact of the end-of-life phase is often uncertain due to differences between what is feasible or intended by the product designer and actual real world practices [10,209]. Until now, the most common waste management approaches for bioplastics were similar to those of conventional plastics: incineration (with or without energy recovery), landfilling, and composting [10,209]. At this point, compostable plastics are not recycled in conventional mechanical recycling plants due to their low quantity. In the future, however, when quantities increase, this may require adaptation of the sorting lines, or generating levels of contamination that may cause problems for mechanical recycling [10].

The dominant end-of-life approach varies by region. For example, in Germany, biodegradable plastics are mostly incinerated (with energy recovery) and composting is uncommon as most of the bioplastics are treated as contaminants in sorting facilities [205]. Because the market share of bioplastics is still small, no specific large-scale sorting or recycling plants were constructed, although mechanical recycling is associated with better carbon footprint results than the other end-of-life scenarios, e.g., recycling PLA causes the emission of 0.62 kg CO_2_ eq./kg PLA compared to around 1.5 kg for composting, 1.6 kg for landfilling, 1.7 kg for incineration, and 2.2 kg for anaerobic digestion [10]. In general, landfilling, incineration, and composting are associated with higher carbon footprints than recycling due to the need for more virgin feedstocks and the conversion of bioplastics into CO_2_ and CH_4_ emissions. However, more research is needed to identify the best waste treatment scenarios for bioplastics as little is known about the technical qualities of secondary bioplastics after one or more recycling cycles [10]. The end-of-life scenario in which bioplastics are fermented and converted into biogas in conventional anaerobic digestion plants needs further research and optimization before large-scale application can be considered feasible [205]. The recuperation of energy in bioplastics due to biogas production via anaerobic digestion is so far limited and needs more studies to standardize and use anaerobic digestion as a valuable end-of-life option.

Land use is another uncertain factor concerning bioplastics: “Is the cultivation of raw materials for bioplastics competing with growing food, other crops such as wood, or nature? Is extra land needed for the expected increase in the production of bioplastics?” [210]. For the impact calculation of Ingeo^®^ PLA, no land change was considered because the arable land had been in use for a long time. This neglects the land still being usable for other purposes. It is assumed that the percentage of the arable land use is very small, even with a 10-fold increase in production capacity [207]. At this moment, the production of bioplastics uses less than 0.02% of the total amount of arable land. Therefore, no important competition with other crops is assumed [211]. The production of biomass feedstock requires more land than fossil fuel extraction, but the impact on the land could have different proportions. In most cases, the impact is less than 10% of the total cradle-to-grave land use impact [209].

Besides the production of the raw materials and their end-of-life fate, material properties and product design also play important roles in the environmental impact of bio-based products. For example, PE waste bags are associated with a lower environmental impact for several impact categories investigated (including climate change) than biodegradable waste bags containing 40–70% fossil raw materials due to the greater thickness of the biodegradable bags. For carrier bags, the standard PE-HD bags have environmental advantages over bioplastic bags composed of a blend of EcoFlex, PLA, and calcium carbonate due to the higher weight of the latter and the CO_2_ and CH_4_ emissions occurring when biodegradable bags are degraded in landfills. PLA pots and cups were compared with PS, PET, and PP counterparts. In this study, the weight and the waste treatment were often different. In most cases, the PLA pots and cups performed better in terms of climate change and fossil resource depletion, and worse for acidification and eutrophication. More variation was found for the other impact categories. For hinged PLA bowls, there were no clear advantages compared to PET, PP, and PS bowls [205].

The European Commission performed a comparative environmental impact evaluation of beverage bottles (bio-based PET compared to petrochemical PET), horticultural clips (starch compared to PP), single-use cups (PLA compared to PP and PET), single-use cutlery (PLA compared to PS), agricultural mulching films (starch compared to LDPE), food packaging films (PLA compared to PP), and single-use carrier bags (starch compared to LDPE). The manufacturing phase accounted for around 50% of the cradle-to-grave impact for all seven case studies. The bioplastics offered environmental benefits for the categories ‘climate change’ and ‘abiotic depletion of fossil fuels’. For ‘particulate matter’, they performed worse. Other categories showed mixed results or were difficult to compare due to high uncertainty. The study showed that the choice of end-of-life scenario has a strong influence on the results, certainly for the carbon footprint, although the results of the end-of-life phase were highly uncertain [209]. The optimization of the production on larger scales of bioplastics, thus comparable with commodities, could also significantly decrease the impact related to their production phase, thus making them more environmentally convenient.

As bioplastics currently represent less than 1% of the total amount of plastics produced globally [211], switching from fossil-fuel- to bio-based plastics could be a step toward meeting the Europe 2020 targets for greenhouse gas emissions [212]. However, only 4% of European greenhouse gas emissions are caused by the use of plastics [210], of which 40% are used for packaging purposes [175,213]. The impact is even lower for European consumers: only 0.6% of their carbon footprint is attributed to plastic packaging materials [210]. So, the potential improvement caused by a switch to bioplastics in terms of global warming is limited. When looking at a wider variety of impact categories, bioplastics are not always environmentally superior to their conventional counterparts but they are competitive in many [205]. However, research into major environmental issues, such as the effects of plastic littering on our environment and health, is not advanced enough to draw conclusions at this stage. Thus, more research is needed to provide the insight required to move toward truly sustainable solutions and bioplastics that outperform conventional materials in the most relevant issues.

### 5.2. Biodegradable Materials

Industrial compostability was the first process of organic recycling for which a test strategy including specific requirements was developed. This resulted in the harmonized EU standard EN 13432: Requirements for packaging recoverable through composting and biodegradation, including a test scheme and evaluation criteria for the final acceptance of packaging, according to which a broad range of products are certified. Examples of plastic materials that can be biodegradable under industrial composting conditions are, for instance, PLA, TPS, PHA, PBS, and PBAT, which can be found on the list of certified products of TÜV AUSTRIA Belgium and DIN CERTCO. Conventional plastics such as LDPE, PP, and PET are not biodegradable by microorganisms within a reasonable time frame and, as such, cannot be treated by composting. However, engineered enzymes have been developed, which might make the microbial degradation of polymers such as PET possible [214].

The biodegradation of a polymer is influenced by the exposure environment (temperature, moisture conditions, microbial population, pH, oxygen content, etc.). A material that degrades by microbial activity under industrial composting conditions does not necessarily degrade in a home composting unit or under aquatic conditions [215]. During home composting, the high temperatures (>50 °C) required by some polymers such as PLA for starting hydrolysis and degradation and obtained during industrial composting processes are mostly not reached. At lower temperatures, the degradation rate is reduced or even limited. Therefore, to be home compostable, a material must demonstrate sufficient biodegradation and disintegration at ambient temperature. This principle was first applied by the OK compost HOME Conformity Mark of certification institute TÜV AUSTRIA Belgium. Currently, the German Certification Institute DIN CERTCO has developed a logo: DIN-Geprüft Home Compostable. At the European level, home composting has become an important factor in France, with the ban on conventional lightweight plastic shopping bags, the encouragement of the use bioplastics compostable at the domestic level, and the introduction of a French national standard on home compostable materials NF T 51-800 Plastics—Specifications for plastic suitable for home composting (2015). At the CEN level, a standard for home compostable carrier bags is being developed by workgroup CEN TC261/SC/WG2 (EN 17427). Several European companies, such as BASF, BIOTEC, LIMAGRAIN, NOVAMONT, FUTAMURA, etc., have developed polymer resins that are home compostable, but they have limited thermomechanical behavior and processability. Most of these products are starch-, polyhydroxy butyrate (PHB)-, or polyhydroxy succinate adipate (PBSA)-based materials, although the Ecovio compounds of BASF contain PLA (in low amounts when meeting home compostability requirements, with the rest being non-bio-based PBAT). On the market, home compostable products meeting the needs of the packaging applications are still lacking (in terms of price, controlled biodegradability, and barrier properties). To achieve low oxygen permeability, co-extruded or laminated multilayer plastic films are widely used in the packaging industry, where mineral-oil-derived EVOH or PVDC are the most-used oxygen barrier materials. However, these polymers are neither biodegradable nor biobased. Therefore, research has recently been performed to increase the possibilities for home compostable packaging. For example, organic coatings have been developed on basis of whey proteins, resulting in higher barrier properties while maintaining the biodegradation [13]. PLA is currently the most affordable bio-based and biodegradable plastic due to the cost and availability on the market, but its biodegradability is not guaranteed under home composting conditions. The development of a PLA that degrades under mild conditions would boost the applications for biodegradable products.

The compostability rate is dependent on thickness. For example, PLA for industrial composting conditions is certified up to 3000 µm thickness (NatureWorks LLC, Minnetonka, MN, USA), but PBS up to 68 µm (Xinjian Blue Ridge Tunhe Polyester Co., Ltd., Changji, China) and polybutylene adipate terephthalate up to 120 µm (BASF, Ludwigshafen, Germany). As biodegradation is also determined by the characteristics of the environment (temperature, moisture content, pH, oxygen presence, and microbial population), specific test procedures were developed to determine biodegradation of plastics under anaerobic conditions (ISO 15985).

Due to the previously mentioned complex range of parameters influencing the actual biodegradation of materials in different conditions, predictive models to support biopolymer selection depending on their target application and end-of life-requirements would be useful.

For predicting the biodegradation of organic compounds (chemicals), different models exist, such as BioWin and BESS.

BioWin (EnviroSim Associates Ltd., Hamilton, ON, Canada) is a “wastewater treatment process simulator that ties together biological, chemical, and physical process models” [216]. BioWin includes a proprietary biological model that “is supplemented with other process models (e.g., water chemistry models for calculation of pH, mass transfer models for oxygen modelling, and other gas-liquid interactions)” [216]. A stock and flow schema are used to represent the system.

BESS [217] is a biodegradability evaluation and simulation system. It can predict the biodegradability of a compound based on the structural features of that compound and the prevailing environmental conditions. It builds hierarchical knowledge based on biodegradation rules, which allow the user to ask questions such as “How does the biodegradation of compound X occur?” The system includes knowledge of first principles on the biodegradation of different structures such as alkanes, amino acids, monocyclic hydrocarbons, and sulfonates, among other categories of structures.

Few state-of-the-art biopolymer biodegradation models are available as it is a much more complex process and a field receiving emerging interest. The publications that do exist, besides those mainly related to experimental data, either avoid modelling or tend to be very specific. For instance, Abi-Akl et al. developed a theoretical model. In this model, the bio-chemo-mechanically coupled kinetics of polymer degradation are assessed. The model includes parameters of the solvent, polymer characteristics, enzyme secretion and diffusion, kinetic parameters, and parameters to describe degradation that are estimated during the modelling process [218]. Another model describing biodegradation proposes a generalized constitutive model for municipal solid waste. The model considers the effects of mechanical creep and time-dependent biodegradation to predict the effect of total landfill compression under incremental loading and with time [219].

### 5.3. Recyclable Materials

The sorting of polymers is of vital importance since even very low amounts of cross-contamination can ruin the entire batch. Material recycling from mixed waste plastics is challenging and based on sophisticated process chains combining different sorting steps like screening, wind sifting, near infrared (NIR) spectroscopic-based sorting, electro-magnetic removal of different metals, and others.

Different technologies able to classify different materials have been developed, and they are already commercially available in the market for most common polymers based on X-ray, color laser, or spectroscopic techniques. An example is Eagle Vision [220], which is able to pre-sort PET, PE, PP, PS, PVC, and PLA using NIR analysis to help remove undesirable plastics from other main streams. In terms of PLA, Titech and Unisensor are commercial examples of NIR systems that are able to discriminate between PET and PLA with accuracies higher than 97%, which is an acceptable amount for maintaining good properties for reprocessed PET [221]. Automatic sorting of other biopolymers, like PHB and starch, or blends is also possible by NIR.

When imaging requirements must be met, as is often the case for plastic sorting, hyperspectral imaging is used. A basic hyperspectral imaging system includes in its set-up a sensitive NIR sensor, a broadband illumination source (tungsten lamps), a spectrometer that separates the backscattered/transmitted light in its different wavelengths, and, when required depending on the configuration (i.e., for push broom configuration), a conveyor belt for sampling. In that case, the conveyor must be synchronized with the recording rate of the CCD sensor for a correct image acquisition. A hyperspectral system provides a hypercube as the output, which is a group of data ordered in three dimensions: two spatial (an XY plane) and one spectral (λ wavelength).

With multilayers, although plastic/plastic composite films are predominately incinerated, they sometimes end up in material flows of different target fractions, e.g., when there is no automated sorting or if the outer layer of a composite is thick enough to let the sorting systems identify the outer layer only. If the density of the composite material is like the density of the target fraction, a further floatation system, which is often in place at recyclers, will not work either. As such, efforts are needed to improve the available systems for multilayer recycling both through the combination of different detection techniques and enhanced data analysis. This could be facilitated using bio-based barrier materials that have very specific spectroscopic signatures compared with other plastics, such as whey protein coatings are easily identified by FTIR [12].

The recycling of bio-based materials consists of effective processing operations producing similar or new products from the materials recovered due to sorting, which is advantageous for increasing the lifespan of the material, as stated previously. Regarding PLA, Cosate de Andrade et al. demonstrated that its recycling has a lower environmental impact than its composting using life cycle assessment studies [222]. Badia et al. reviewed PLA recycling [223]. The degradation of PLA was discussed as it can occur during recycling when recovered plastics are reprocessed by extrusion. Hydrolysis due to water and thermal degradation was considered. Reprocessing of PLA can additionally induce an increase in crystallization during cooling with the number of injection cycles due to chain scission during injection. The hydrolysis of PLA can be limited to drying. The use of chain extenders was reported to be useful for controlling the melt viscosity of PLA and its blends, as shown by Najafi et al., who evidenced the effectiveness of three different chain extenders and clay at 2% in an attempt to control the melt viscosity [224]. For mechanical recycling of biopolyesters, an uncontaminated starting material is essential. Contamination can occur due to additives blended in the biopolyester matrix or layers. In the former case, the compatibility between the PLA matrix and the additive is generally achieved. Conversely, when a different layer is present, successive processing must include a compatibilization strategy, as when using copolymers as the compatibilizer in PLA/cellulose composites [225] or cellulose esters in wood/poly(lactic acid) composites [226]. In blends of different polyesters, the use of radical agents [227] or transesterification catalysts [228] was studied as possible reactive processing methodologies for improving compatibility.

The recycling of monomaterial PLA via grinding of sheets and trays, washing of the scraps, drying, and processing in an extrusion machine to obtain pellets was also studied in PLA4FOOD and BIO4MAP projects. The mechanical and rheological results revealed that the flowability of the melt and the mechanical properties of the materials produced are maintained during several processing cycles, which assure the possibility of using recycled PLA in blends with virgin PLA without the loss of material characteristics.

In the case of polymer-based multilayers, several methods can be used to separate the layers either by delamination or by selective dissolution–reprecipitation methods, which were extensively reviewed elsewhere [8].

Due to its low depolymerization temperature and possible presence of impurities, as an alternative to mechanical recycling, PLA is a good candidate for environmentally sound chemical recycling as it can be readily hydrolyzed with water to form LA, which is then purified and polymerized to remake prime PLA [229]. Piemonte et al. demonstrated, through a life cycle assessment approach, that the production of LA from chemical depolymerization of PLA is preferable, from an energy point of view, than the production of LA by glucose fermentation [230].

## 6. Conclusions and Perspectives

This review paper summarizes the current state of bio-based and biodegradable polymers as well as the different related processes used in packaging with a tailored end of life mainly consisting of biodegradation and recycling. More than 200 sources were reviewed by different academic and industry bioplastic experts, with complementary studies along the value chain including scientific literature, patents, and commercial information.

We focused on bio-based copolymers and compounds with biocomposites, coatings, and surface modifications to tailor packaging properties. In terms of processes, the different steps to produce rigid and flexible packaging including textiles were described. The use of bio-based virgin or recycled materials into market-available packaging was also reported. The environmental impacts of these materials were discussed, providing insight into the available end-of-life options, progress, and challenges.

In conclusion, this review shows that the bio-based polymers belonging to the category of polyesters are generally slow to biodegrade; hence, the modification of their structure through copolymerization or other techniques can be convenient for modulating this property, increasing the speed and ease of their biodegradation. These efforts can lead to the production of materials that are biodegradable in mild conditions, for instance, in home composting conditions or in soil.

For non-synthetic materials such as polysaccharides and proteins, they generally biodegrade quickly; hence, they can be considered useful processing aids, fillers, or modifiers for biopolyesters. They can contribute to improving their properties (barrier, mechanical, and rheological properties) for making them suitable for specific packaging applications. However, the effects of their presence on the recyclability of biopolyesters require specific technical and scientific studies.

Improving the recyclability of biopolyesters-based materials can be advantageous for postponing their final biodegradation, thus avoiding the production of corresponding raw materials, and decreasing their environmental impact.

Given the “European Strategy for Plastics in a Circular Economy” released in 2018 and other related emerging policies, new packaging materials must be provided and adopted to attain the holistic sustainability of the value chain. This review highlighted the increasing number of investigations and the increasing industrial interest in the area as well as further developments needed.

## Figures and Tables

**Table 1:** Summary of the main characteristics of bio-based materials for packaging and textile applications.

Bio-Based Material	Synthetic/Non-Synthetic Produced Polymer	Classification	Common Feedstock or Source	Monomer/Sub-Unit	Common Production Technique	Main Intrinsic Variables	Expected Production Capacity by 2023 (Million Tonnes) [29]	End of Life (Common)	Recent Investigations in the Field of Packaging	References
Polylactic Acid (PLA)	Synthetic	Aliphatic Polyester	Corn, Corn Stover, Sugarcane Bagasse, Sugar Beet, Rice Hulls	Lactic Acid (L- and D-Isomers)	Ring Opening Polymerisation Technique; Polycondensation	Ratio of Isomers; Molecular Weight	0.83	Industrial Composting, Mechanical and Chemical recy Cling	CopolymerizAtion with Dicarboxylic Acids; Degradation Accelerating Modifications or Additives; Blending PLA	[30,31,32,33,34]
Polyethylene Furanoate (PEF)	Synthetic	Aromatic Polyester	Corn, Wheat	2,5-FurandicarboXylic Acid, Monoethylene Glycol	Polycondensation	Molecular Weight	No Data	Target Recycling when Market Grows	Alternative Feedstocks: Techniques to Lower the price	[35,36]
Polybutylene Succinate (PBS)	Synthetic	Aliphatic Polyester	Sugar Cane, Sugar Beet, Corn, Potato, Wheat	Succinic Acid and 1,4-Butanediol,	Polyconcensation	Molecular Weight	0.54 (Includes PBS, PBAT and PCL)	Chemical/Catalytic Recycling, Enzymatic Depolymerisation	PBS in Composites to Enhance Processability	[29,37,38,39,40,41]
PolyhydroxyalkaNoate (PHA)	Non-Synthetic	Aliphatic Polyester (Family)	Sugars and Emerging Trials with Waste Biomass	Depending on the Sub Type	Microbial Fermentation	Molecular Weight; Monomer Types; Crystallinity	0.17	Industrial and Home Composting; Biogas Installation	Reduction of Production Costs (Mixed Microbial Cultures; Low Cost Feedstocks; Fillers or Blends)	[9,25,41,42,43,44]
Cellulose	Non-Synthetic	Polysaccharide	Plant material	β-D-Glucose	Naturally Occurring	Regenerated Cellulose	0.03	Home composting, Industrial Composting, Anaerobic Digestion;	Cellulose Nanocrystal and Cellulose Nanofiber Films and Composites	[41,45,46,47]
Starch	Non-Synthetic	Polysaccharide	Potatoes, Corn, Wheat, Rice, Tapioca	D-Glucose	Naturally Occurring	Thermoplastic Starch	0.22	Totally Biodegradabl; Industrial or Home Compost	Starch in Composite Films	[41,48,49,50,51]
Protein	Non-Synthetic	Proteins	Plants and Animals	Amino Acids	Naturally Occurring	Amino Acid Composition, Source; Degree of Denaturation; Purity	No Data	Totally Biodegradable	Physical, Chemical, and Enzymatic Modifications of Protein Films and Coatings	[12,13,15,16,20]
Lipids and Waxes	Non-Synthetic	Lipids	Plants and Animals	Fatty Acids and Other Hydrocarbons	Naturally Occurring	Carbon Length of Fatty Acid; Source; Accompanying Substances	No Data	Totally Biodegradable	Edible Coatings; Multilayer	[31,52,53]

## References

[1] Coltelli M.-B., Gigante V., Cinelli P., Lazzeri A., Morganti P. (2018). Chapter 15 Flexible Food Packaging Using Polymers from Biomass. Bionanotechnology to Save the Environment; Plant and Fishery’s Biomass as Alternative to Petrol.

[2] Álvarez-Chávez C.R., Edwards S., Moure-Eraso R., Geiser K. (2012). Sustainability of bio-based plastics: General comparative analysis and recommendations for improvement. J. Clean. Prod..

[3] Prashanth K.H., Tharanathan R. (2007). Chitin/Chitosan: Modifications and their unlimited application potential—An overview. Trends Food Sci. Technol..

[4] European Bioplastics Bioplastics Market Data. https://www.european-bioplastics.org/.

[5] Bajpai P. (2019). Biobased Polymers. Properties and Applications in Packaging.

[6] Amcor 2018 Sustainability Review. https://www.amcor.com/sustainability/reports.

[7] Geueke B., Groh K., Muncke J. (2018). Food packaging in the circular economy: Overview of chemical safety aspects for commonly used materials. J. Clean. Prod..

[8] Kaiser K.M.A., Schmid M., Schlummer M. (2017). Recycling of Polymer-Based Multilayer Packaging: A Review. Recycling.

[9] Thielen M. (2012). Bioplastics: Basics, Applications, Markets.

[10] Spierling S., Knüpffer E., Behnsen H., Mudersbach M., Krieg H., Springer S., Albrecht S., Herrmann C., Endres H.-J. (2018). Bio-based plastics—A review of environmental, social and economic impact assessments. J. Clean. Prod..

[11] Lee R., Lavoie J.-M. (2013). From first- to third-generation biofuels: Challenges of producing a commodity from a biomass of increasing complexity. Anim. Front..

[12] Bugnicourt E., Schmid M., Nerney O.M., Wildner J., Smykala L., Lazzeri C., Cinelli P. (2013). Processing and Validation of Whey-Protein-Coated Films and Laminates at Semi-Industrial Scale as Novel Recyclable Food Packaging Materials with Excellent Barrier Properties. Adv. Mater. Sci. Eng..

[13] Cinelli P., Schmid M., Bugnicourt E., Wildner J., Bazzichi A., Anguillesi I., Lazzeri C. (2014). Whey protein layer applied on biodegradable packaging film to improve barrier properties while maintaining biodegradability. Polym. Degrad. Stab..

[14] Schmid M., Benz A., Stinga C., Samain D., Zeyer K.P. (2012). Fundamental Investigations Regarding Barrier Properties of Grafted PVOH Layers. Int. J. Polym. Sci..

[15] Schmid M., Dallmann K., Bugnicourt E., Cordoni D., Wild F., Lazzeri C., Noller K. (2012). Properties of Whey-Protein-Coated Films and Laminates as Novel Recyclable Food Packaging Materials with Excellent Barrier Properties. Int. J. Polym. Sci..

[16] Zink J., Wyrobnik T., Prinz T., Schmid M. (2016). Physical, Chemical and Biochemical Modifications of Protein-Based Films and Coatings: An Extensive Review. Int. J. Mol. Sci..

[17] Schmid M., Müller K., Deeth H.C., Bansal N. (2019). Chapter 11—Whey Protein-Based Packaging Films and Coatings.

[18] Hong S.-I., Krochta J. (2006). Oxygen barrier performance of whey-protein-coated plastic films as affected by temperature, relative humidity, base film, and protein type. J. Food Eng..

[19] Zhou J., Wang S., Gunasekaran S. (2009). Preparation and Characterization of Whey Protein Film Incorporated with TiO2 Nanoparticles. J. Food Sci..

[20] Ustunol Z., Mert B. (2006). Water Solubility, Mechanical, Barrier, and Thermal Properties of Cross-linked Whey Protein Isolate-based Films. J. Food Sci..

[21] Samain D. (2002). Method for Treating a Solid Material to Make It Hydrophobic, Material Obtained and Uses.

[22] Chang Y., Joo E., Song H.-G., Choi I., Yoon C.S., Choi Y.J., Han J. (2019). Development of Protein-Based High-Oxygen Barrier Films Using an Industrial Manufacturing Facility. J. Food Sci..

[23] Hu B. (2014). Biopolymer-Based Lightweight Materials for Packaging Applications. ACS Symp. Series.

[24] Terzopoulou Z., Tsanaktsis V., Bikiaris D.N., Exarhopoulos S., Papageorgiou D. (2016). Biobased poly(ethylene furanoate-co-ethylene succinate) copolyesters: Solid state structure, melting point depression and biodegradability. RSC Adv..

[25] Camebridge Consultants. PHA: Plastic the Way Nature Intendend?. https://www.cambridgeconsultants.com/insights/bioplastics-pha-whitepaper.

[26] Stloukal P., Pekařová S., Kalendova A., Mattausch H., Laske S., Holzer C., Chitu L., Bodner S., Maier G., Slouf M. (2015). Kinetics and mechanism of the biodegradation of PLA/clay nanocomposites during thermophilic phase of composting process. Waste Manag..

[27] Padamati R.B., O’Connor K.E., Ramakrishna S. (2013). Current progress on bio-based polymers and their future trends. Prog. Biomater..

[28] Blackburn R.S. (2009). Sustainable Textiles. Life Cycle and Environmental Impact.

[29] IfBB Institute for Bioplastics and Biocomposites (2019). Biopolymers Facts and Statistics: Production Capacities, Processing Routes, Feedstock, Land and Water Use (ISSN (Online) 2510-3431).

[30] Singhvi M., Gokhale D. (2013). Biomass to biodegradable polymer (PLA). RSC Adv..

[31] Hanstveit A.O. (1992). Biodegradability of petroleum waxes and beeswax in an adapted CO2 evolution test. Chemosphere.

[32] Auras R., Lim L.-T., Selke S.E.M., Tsuji H. (2010). Poly(lactic acid). Synthesis, Structures, Properties, Processing, and Applications.

[33] Garlotta D. (2001). A Literature Review of Poly(Lactic Acid). J. Polym. Environ..

[34] Perinelli D.R., Cespi M., Bonacucina G., Palmieri G.F. (2019). PEGylated polylactide (PLA) and poly(lactic-co-glycolic acid) (PLGA) copolymers for the design of drug delivery systems. J. Pharm. Investig..

[35] Eerhart A.J.J.E., Faaij A.P.C., Patel M.K. (2012). Replacing fossil-based PET with biobased PEF; Process analysis, energy and GHG balance. Energy Environ. Sci..

[36] Avantium Internal Results YXY Technologies. https://www.avantium.com/technologies/yxy/.

[37] Xu J., Guo B.-H. (2009). Microbial Succinic Acid, Its Polymer Poly(butylene succinate), and Applications. Prokaryotic Symbionts in Plants.

[38] Guettler M.V., Jain M.K., Rumler D. (1995). Method for Making Succinic Acid, Bacterial Variants for Use in Process and Methods for Obtaining Variants. U.S. Patent.

[39] Jompang L., Thumsorn S., On J.W., Surin P., Apawet C., Chaichalermwong T., Kaabbuathong N., O-Charoen N., Srisawat N. (2013). Poly(Lactic Acid) and Poly(Butylene Succinate) Blend Fibers Prepared by Melt Spinning Technique. Energy Proc..

[40] Seggiani M., Gigante V., Cinelli P., Coltelli M.-B., Sandroni M., Anguillesi I., Lazzeri A. (2019). Processing and mechanical performances of Poly(Butylene Succinate–co–Adipate) (PBSA) and raw hydrolyzed collagen (HC) thermoplastic blends. Polym. Test..

[41] Rameshkumar S., Shaiju P., O’Connor K.E., Padamati R.B. (2020). Bio-based and biodegradable polymers—State-of-the-art, challenges, and emerging trends. Curr. Opin. Green Sustain. Chem..

[42] Sabapathy P.C., Devaraj S., Meixner K., Anburajan P., Kathirvel P., Ravikumar Y., Zabed H.M., Qi X. (2020). Recent developments in Polyhydroxyalkanoates (PHAs) production—A review. Bioresour. Technol..

[43] Bugnicourt E. (2014). Polyhydroxyalkanoate (PHA): Review of synthesis, characteristics, processing, and potential applications in packaging. Express Polym. Lett..

[44] Dilkes-Hoffman L.S., Lant P.A., Laycock B., Pratt S. (2019). The rate of biodegradation of PHA bioplastics in the marine environment: A meta-study. Mar. Pollut. Bull..

[45] Mishra R.K., Ha S.K., Verma K., Tiwari S.K., Kumar R. (2018). Recent progress in selected bio-nanomaterials and their engineering applications: An overview. J. Sci. Adv. Mater. Devices.

[46] Pandey J.K., Kumar A.P., Misra M., Mohanty A.K., Drzal L.T., Singh R.P. (2005). Recent advances in biodegradable nanocomposites. J. Nanosci. Nanotechnol..

[47] Endres H.-J., Siebert-Raths A. (2011). Engineering Biopolymers. Markets, Manufacturing, Properties, and Applications.

[48] Johansson C., Bras J., Mondragon I., Nechita P., Plackett D., Simon P., Svetec D.G., Virtanen S., Baschetti M.G., Breen C. (2012). Renewable fibers and bio-based materials for packaging applications-a review of recent developments. Bioresources.

[49] Lawton J., Fanta G. (1994). Glycerol-plasticized films prepared from starch—poly(vinyl alcohol) mixtures: Effect of poly(ethylene-co-acrylic acid). Carbohydr. Polym..

[50] Imam S.H., Gordon S.H., Shogren R.L., Tosteson T.R., Govind N.S., Greene R.V. (1999). Degradation of Starch–Poly(β-Hydroxybutyrate-Co-β-Hydroxyvalerate) Bioplastic in Tropical Coastal Waters. Appl. Environ. Microbiol..

[51] Bertolini A. (2010). Starches. Characterization, Properties, and Applications.

[52] Callegarin F., Gallo J.-A.Q., Debeaufort F., Voilley A. (1997). Lipids and biopackaging. J. Am. Oil Chem. Soc..

[53] Dhall R.K. (2013). Advances in Edible Coatings for Fresh Fruits and Vegetables: A Review. Crit. Rev. Food Sci. Nutr..

[54] Castro-Aguirre E., Iñiguez-Franco F., Samsudin H., Fang X., Auras R. (2016). Poly(lactic acid)—Mass production, processing, industrial applications, and end of life. Adv. Drug Deliv. Rev..

[55] Mehta R., Kumar V., Bhunia H., Upadhyay S.N. (2005). Synthesis of Poly(Lactic Acid): A Review. J. Macromol. Sci. Part C.

[56] Ren J. (2011). Biodegradable Poly(Lactic Acid): Synthesis, Modification, Processing and Applications.

[57] Li B., Chen S.-C., Qiu Z.-C., Yang K.-K., Tang S., Yu W.-J., Wang Y. (2008). Synthesis of poly(lactic acid-b-p-dioxanone) block copolymers from ring opening polymerization of p-dioxanone by poly(L-lactic acid) macroinitiators. Polym. Bull..

[58] Ovitt T.M., Coates G.W. (2000). Stereoselective ring-opening polymerization of rac-lactide with a single-site, racemic aluminum alkoxide catalyst: Synthesis of stereoblock poly(lactic acid). J. Polym. Sci. Part A Polym. Chem..

[59] Radano C.P., Baker G.L., Smith M.R. (2000). Stereoselective Polymerization of a Racemic Monomer with a Racemic Catalyst: Direct Preparation of the Polylactic Acid Stereocomplex from Racemic Lactide. J. Am. Chem. Soc..

[60] NatureWorks Where Ingeo Comes From. https://www.natureworksllc.com/.

[61] Raquez J.-M., Ramy-Ratiarison R., Murariu M., Dubois P. (2014). CHAPTER 4. Reactive Extrusion of PLA-based Materials: From Synthesis to Reactive Melt-blending. Polymer Chem. Series.

[62] Dugan J.S. (2001). Novel Properties of PLA Fibers. Int. Nonwovens J..

[63] Fazita M.N., Jayaraman K., Bhattacharyya D., Haafiz M.M., Saurabh C., Hussin M.H., Khalil H.A. (2016). Green Composites Made of Bamboo Fabric and Poly(Lactic) Acid for Packaging Applications—A Review. Materials.

[64] Maharana T., Mohanty B., Negi Y.S. (2009). Melt–solid polycondensation of lactic acid and its biodegradability. Prog. Polym. Sci..

[65] Lyu S., Schley J., Loy B., Lind D., Hobot C., Sparer R., Untereker D. (2007). Kinetics and Time−Temperature Equivalence of Polymer Degradation. Biomacromolecules.

[66] El Sawy M., Kim K.-H., Park J.-W., Deep A. (2017). Hydrolytic degradation of polylactic acid (PLA) and its composites. Renew. Sustain. Energy Rev..

[67] Husarova L., Pekařová S., Stloukal P., Kucharzcyk P., Verney V., Commereuc S., Ramoné A., Koutný M. (2014). Identification of important abiotic and biotic factors in the biodegradation of poly(l-lactic acid). Int. J. Boil. Macromol..

[68] Mueller R.-J. (2006). Biological degradation of synthetic polyesters—Enzymes as potential catalysts for polyester recycling. Process. Biochem..

[69] Kijchavengkul T., Auras R., Rubino M., Ngouajio M., Fernandez R.T. (2008). Assessment of aliphatic–aromatic copolyester biodegradable mulch films. Part II: Laboratory simulated conditions. Chemosphere.

[70] Gorrasi G., Pantani R. (2013). Effect of PLA grades and morphologies on hydrolytic degradation at composting temperature: Assessment of structural modification and kinetic parameters. Polym. Degrad. Stab..

[71] Ghorpade V.M., Gennadios A., Hanna M.A. (2001). Laboratory composting of extruded poly(lactic acid) sheets. Bioresour. Technol..

[72] Marty A., Duquesne S., Guicherd M., Gueroult M., Andre I. (2019). Novel Proteases and Uses Therof.

[73] Tokiwa Y., Jarerat A. (2004). Biodegradation of poly(L-lactide). Biotechnol. Lett..

[74] Blasi P. (2019). Poly(lactic acid)/poly(lactic-co-glycolic acid)-based microparticles: An overview. J. Pharm. Investig..

[75] Sharma D., Lipp L., Arora S., Singh J. (2019). Diblock and triblock copolymers of polylactide and polyglycolide. Mater. Biomed.Eng..

[76] Koosomsuan W., Yamaguchi M., Phinyocheep P., Sirisinha K. (2018). High-Strain Shape Memory Behavior of PLA-PEG Multiblock Copolymers and Its Microstructural Origin. J. Polym. Sci. Part B Polym. Phys..

[77] Liu X., Corciulo C., Arabagian S., Ulman A., Cronstein B. (2019). Adenosine-Functionalized Biodegradable PLA-b-PEG Nanoparticles Ameliorate Osteoarthritis in Rats. Sci. Rep..

[78] Lee G.S., Moon B.R., Jeong H., Shin J., Kim J.G. (2019). Mechanochemical synthesis of poly(lactic acid) block copolymers: Overcoming the miscibility of the macroinitiator, monomer and catalyst under solvent-free conditions. Polym. Chem..

[79] Werpy T., Petersen G. (2004). Top Value-Added Chemicals from Biomass: Volume I—Results of Screening for Potential Candidates from Sugars and Synthesis Gas (technical report).

[80] Thessrimuang N., Prachayawarakorn J. (2018). Characterization and Properties of High Amylose Mung Bean Starch Biodegradable Films Cross-linked with Malic Acid or Succinic Acid. J. Polym. Environ..

[81] Cheng C., Zhou Y., Lin M., Wei P., Yang S.-T. (2017). Polymalic acid fermentation by Aureobasidium pullulans for malic acid production from soybean hull and soy molasses: Fermentation kinetics and economic analysis. Bioresour. Technol..

[82] Iyyappan J., Bharathiraja B., Baskar G., Kamalanaban E. (2019). Process optimization and kinetic analysis of malic acid production from crude glycerol using Aspergillus niger. Bioresour. Technol..

[83] Gazzotti S., Todisco S.A., Picozzi C., Ortenzi M.A., Farina H., Lesma G., Silvani A. (2019). Eugenol-grafted aliphatic polyesters: Towards inherently antimicrobial PLA-based materials exploiting OCA’s chemistry. Eur. Polym. J..

[84] Zhang Y., Ni C., Shi G., Wang J., Zhang M., Li W. (2014). The polyion complex nano-prodrug of doxorubicin (DOX) with poly(lactic acid-co-malic acid)-block-polyethylene glycol: Preparation and drug-controlled release. Med. Chem. Res..

[85] Oyama H.T., Tanishima D., Maekawa S. (2016). Poly(malic acid-co-L-lactide) as a superb degradation accelerator for Poly(l-lactic acid) at physiological conditions. Polym. Degrad. Stab..

[86] Ferone M., Raganati F., Olivieri G., Marzocchella A. (2019). Bioreactors for succinic acid production processes. Crit. Rev. Biotechnol..

[87] Su S., Kopitzky R., Tolga S., Kabasci S. (2019). Polylactide (PLA) and Its Blends with Poly(butylene succinate) (PBS): A Brief Review. Polymer.

[88] Cadar O., Paul M., Roman C., Miclean M., Majdik C. (2012). Biodegradation behaviour of poly(lactic acid) and (lactic acid-ethylene glycol-malonic or succinic acid) copolymers under controlled composting conditions in a laboratory test system. Polym. Degrad. Stab..

[89] Robert T., Friebel S. (2016). Itaconic acid—A versatile building block for renewable polyesters with enhanced functionality. Green Chem..

[90] Sood S., Gupta V.K., Agarwal S., Dev K., Pathania D. (2017). Controlled release of antibiotic amoxicillin drug using carboxymethyl cellulose-cl-poly(lactic acid-co-itaconic acid) hydrogel. Int. J. Boil. Macromol..

[91] Gupta V.K., Sood S., Agarwal S., Saini R.V., Pathania D. (2018). Antioxidant activity and controlled drug delivery potential of tragacanth gum-cl- poly(lactic acid-co-itaconic acid) hydrogel. Int. J. Boil. Macromol..

[92] Aliotta L., Cinelli P., Coltelli M.B., Righetti M.C., Gazzano M., Lazzeri C. (2017). Effect of nucleating agents on crystallinity and properties of poly(lactic acid) (PLA). Eur. Polym. J..

[93] Castiello S., Coltelli M.-B., Bronco S., Conzatti L. (2012). Comparative study about preparation of poly(lactide)/Organophilic montmorillonites nanocomposites through melt blending or ring opening polymerization methods. J. Appl. Polym. Sci..

[94] Scatto M., Salmini E., Castiello S., Coltelli M.-B., Conzatti L., Stagnaro P., Andreotti L., Bronco S. (2012). Plasticized and nanofilled poly(lactic acid)-based cast films: Effect of plasticizer and organoclay on processability and final properties. J. Appl. Polym. Sci..

[95] Shogren R. (1997). Water vapor permeability of biodegradable polymers. J. Polym. Environ..

[96] Singh A.A., Sharma S., Srivastava M., Majumdar A. (2020). Modulating the properties of polylactic acid for packaging applications using biobased plasticizers and naturally obtained fillers. Int. J. Boil. Macromol..

[97] Jem K.J., Tan B. (2020). The development and challenges of poly(lactic acid) and poly(glycolic acid). Adv. Ind. Eng. Polym. Res..

[98] Burgess S.K., Karvan O., Johnson J., Kriegel R.M., Koros W.J. (2014). Oxygen sorption and transport in amorphous poly(ethylene furanoate). Polymer.

[99] Poulopoulou N., Kasmi N., Siampani M., Terzopoulou Z.N., Bikiaris D.N., Achilias D.S., Papageorgiou D.G., Papageorgiou G.Z. (2019). Exploring Next-Generation Engineering Bioplastics: Poly(alkylene furanoate)/Poly(alkylene terephthalate) (PAF/PAT) Blends. Polymers.

[100] Materials E.E.P.O.F.C. (2014). Scientific Opinion on the safety assessment of the substance, furan 2, 5? Dicarboxylic acid, CAS No 3238?40? 2, for use in food contact materials. EFSA J..

[101] Chen G.-Q. (2009). A microbial polyhydroxyalkanoates (PHA) based bio- and materials industry. Chem. Soc. Rev..

[102] Nagarajan V., Andrzejewski J., Misra M. (2016). Perspective on Polylactic Acid (PLA) based Sustainable Materials for Durable Applications: Focus on Toughness and Heat Resistance. ACS Sustain. Chem. Eng..

[103] van den Oever M., Molenveld K., van der Zee M., Bos H. (2017). Bio-Based and Biodegradable Plastics—Facts and Figures.

[104] Danimer Scientific PHA Impact. https://danimerscientific.com/pha-the-future-of-biopolymers/.

[105] Coltelli M.-B., Danti S., Trombi L., Morganti P., Donnarumma G., Baroni A., Fusco A., Lazzeri C. (2018). Preparation of Innovative Skin Compatible Films to Release Polysaccharides for Biobased Beauty Masks. Cosmetics.

[106] Jost V. (2018). Packaging related properties of commercially available biopolymers—An overview of the status quo. Express Polym. Lett..

[107] Yang H.-S., Gardner D.J., Nader J.W. (2010). Dispersion evaluation of microcrystalline cellulose/cellulose nanofibril-filled polypropylene composites using thermogravimetric analysis. J. Therm. Anal. Calorim..

[108] Chen J., Sinclair R. (2015). Chapter 4—Synthetic Textile Fibers: Regenerated Cellulose Fibers. Textiles and Fashion: Woodhead Publishing Series in Textiles.

[109] Weller I., Lange H. (2007). Nachhaltigkeit. Soziologische Rev..

[110] Calva-Estrada S.J., Jiménez-Fernández M., Lugo-Cervantes E. (2019). Protein-Based Films: Advances in the Development of Biomaterials Applicable to Food Packaging. Food Eng. Rev..

[111] Wheylayer 2. http://www.wheylayer.eu/de/.

[112] ThermoWhey. http://thermowhey.eu/.

[113] Stäbler A., Schmid M. (2016). Thermoformbarer Mehrschichtverbund Sowie Proteinbasierte Formulierung zum Erhalt Einer Thermoformbaren Schicht mit Sauerstoffbarriere im Verbund. Patent application.

[114] Bugnicourt E., Noller K., Schmid M., Wild F. (2013). Whey Protein Coated Films; 2011.

[115] Corso J.F. (1963). Bone-Conduction Thresholds for Sonic and Ultrasonic Frequencies. J. Acoust. Soc. Am..

[116] Kadam D.M., Thunga M., Wang S., Kessler M., Grewell D., Lamsal B.P., Yu C. (2013). Preparation and characterization of whey protein isolate films reinforced with porous silica coated titania nanoparticles. J. Food Eng..

[117] Fachin L., Viotto W.H. (2005). Effect of pH and heat treatment of cheese whey on solubility and emulsifying properties of whey protein concentrate produced by ultrafiltration. Int. Dairy J..

[118] Nicorescu I., Loisel C., Vial C., Riaublanc A., Djelveh G., Cuvelier G., Legrand J. (2008). Combined effect of dynamic heat treatment and ionic strength on the properties of whey protein foams—Part II. Food Res. Int..

[119] Nicolai T., Britten M., Schmitt C. (2011). β-Lactoglobulin and WPI aggregates: Formation, structure, and applications. Food Hydrocoll..

[120] Jambrak A.R., Lelas V., Mason T.J., Krešić G., Badanjak M. (2009). Physical properties of ultrasound treated soy proteins. J. Food Eng..

[121] Weng W., Zheng H. (2015). Effect of transglutaminase on properties of tilapia scale gelatin films incorporated with soy protein isolate. Food Chem..

[122] di Pierro P., Chico B., Villalonga R., Mariniello L., Damiao A.E., Masi P., Porta R. (2006). Chitosan−Whey Protein Edible Films Produced in the Absence or Presence of Transglutaminase: Analysis of Their Mechanical and Barrier Properties. Biomacromolecules.

[123] Hernàndez-Balada E., Taylor M.M., Phillips J.G., Marmer W.N., Brown E.M. (2009). Properties of biopolymers produced by transglutaminase treatment of whey protein isolate and gelatin. Bioresour. Technol..

[124] Banerjee R., Chen H. (1995). Functional Properties of Edible Films Using Whey Protein Concentrate. J. Dairy Sci..

[125] Dangaran K., Krochta J., Onwulata C., Huth P.J. (2009). Whey Protein Films and Coatings (chapter 6); Whey Processing, Functionality and Health Benefits.

[126] Fornes T., Paul D.R. (2003). Modeling properties of nylon 6/clay nanocomposites using composite theories. Polymer.

[127] Zeng Q., Yu A., Lu G., Paul D.R. (2005). Clay-Based Polymer Nanocomposites: Research and Commercial Development. J. Nanosci. Nanotechnol..

[128] Kumar P., Sandeep K., Alavi S., Truong V., Gorga R. (2010). Effect of Type and Content of Modified Montmorillonite on the Structure and Properties of Bio-Nanocomposite Films Based on Soy Protein Isolate and Montmorillonite. J. Food Sci..

[129] Kumar P., Sandeep K., Alavi S., Truong V.-D., Gorga R. (2010). Preparation and characterization of bio-nanocomposite films based on soy protein isolate and montmorillonite using melt extrusion. J. Food Eng..

[130] Li Y., Jiang Y., Liu F., Ren F., Zhao G., Leng X. (2011). Fabrication and characterization of TiO2/whey protein isolate nanocomposite film. Food Hydrocoll..

[131] Pérez-Gago M.B., Krochta J.M. (2001). Lipid particle size effect on water vapor permeability and mechanical properties of whey protein/beeswax emulsion films. J. Agric. Food Chem..

[132] Simelane S., Ustunol Z. (2005). Mechanical Properties of Heat? Cured Whey Protein? Based Edible Films Compared with Collagen Casings under Sausage Manufacturing Conditions. J. Food Sci..

[133] Rhim J.W., Gennadios A., Fu D., Weller C.L., Hanna M.A. (1999). Properties of Ultraviolet Irradiated Protein Films. LWT.

[134] Gennadios A., Rhim J., Handa A., Weller C., Hanna M. (2008). Ultraviolet Radiation Affects Physical and Molecular Properties of Soy Protein Films. J. Food Sci..

[135] Morillon V., Debeaufort F., Blond G., Capelle M., Voilley A. (2002). Factors Affecting the Moisture Permeability of Lipid-Based Edible Films: A Review. Crit. Rev. Food Sci. Nutr..

[136] Ashok A., Rejeesh C.R., Renjith R. (2016). Biodegradable Polymers for Sustainable Packaging Applications: A Review. Int. J. Bionics Biomater..

[137] Bravin B., Peressini D., Sensidoni A. (2006). Development and application of polysaccharide–lipid edible coating to extend shelf-life of dry bakery products. J. Food Eng..

[138] Shellhammer T., Krochta J. (1997). Whey Protein Emulsion Film Performance as Affected by Lipid Type and Amount. J. Food Sci..

[139] European Bioplastics What Are Bioplasctics?. https://www.european-bioplastics.org/bioplastics/.

[140] European Commission Sustainable Development. https://ec.europa.eu/trade/policy/policy-making/sustainable-development/.

[141] Wayan S.I., Suriadi I.G.A.K., Arnis K. (2014). Mechanical Properties of Rice Husks Fiber Reinforced Polyester Composites. Int. J. Mater. Mech. Manuf..

[142] Schmid M., Herbst C., Müller K., Stäbler A., Schlemmer D., Coltelli M.-B., Lazzeri C. (2015). Effect of Potato Pulp Filler on the Mechanical Properties and Water Vapor Transmission Rate of Thermoplastic WPI/PBS Blends. Polym. Technol. Eng..

[143] Baştürka S.B., Kanbur K., Polatoğluc I., Yüreklid Y. (2015). Mechanical Properties of Acorn and Pinecone Filled Polymer Composites. Am. Sci. Res. J. Eng. Technol. Sci..

[144] Bledzki A., Gassan J. (1999). Composites reinforced with cellulose based fibres. Prog. Polym. Sci..

[145] Faruk O., Bledzki A.K., Fink H.-P., Sain M. (2012). Biocomposites reinforced with natural fibers: 2000–2010. Prog. Polym. Sci..

[146] Pandey J.K., Ahn S.H., Lee C.S., Mohanty A.K., Misra M. (2010). Recent Advances in the Application of Natural Fiber Based Composites. Macromol. Mater. Eng..

[147] Poulose A.M., Elnour A.Y., Anis A., Shaikh H., Al-Zahrani S., George J., Al-Wabel M.I., Usman A.R., Ok Y.S., Tsang D.C. (2017). Date palm biochar-polymer composites: An investigation of electrical, mechanical, thermal and rheological characteristics. Sci. Total. Environ..

[148] Zhang Q., Zhang D., Xu H., Lu W., Ren X., Cai H., Lei H., Huo E., Zhao Y., Qian M. (2020). Biochar filled high-density polyethylene composites with excellent properties: Towards maximizing the utilization of agricultural wastes. Ind. Crop. Prod..

[149] Aliotta L., Gigante V., Coltelli M.-B., Cinelli P., Lazzeri C., Seggiani M. (2019). Thermo-Mechanical Properties of PLA/Short Flax Fiber Biocomposites. Appl. Sci..

[150] Righetti M.C., Cinelli P., Mallegni N., Stäbler A., Lazzeri C. (2019). Thermal and Mechanical Properties of Biocomposites Made of Poly(3-hydroxybutyrate-co-3-hydroxyvalerate) and Potato Pulp Powder. Polymer.

[151] Coltelli M.-B., Cinelli P., Gigante V., Aliotta L., Morganti P., Panariello L., Lazzeri C. (2019). Chitin Nanofibrils in Poly(Lactic Acid) (PLA) Nanocomposites: Dispersion and Thermo-Mechanical Properties. Int. J. Mol. Sci..

[152] Coltelli M.-B., Aliotta L., Vannozzi A., Morganti P., Panariello L., Danti S., Neri S., Fernandez-Avila C., Fusco A., Donnarumma G. (2020). Properties and Skin Compatibility of Films Based on Poly(Lactic Acid) (PLA) Bionanocomposites Incorporating Chitin Nanofibrils (CN). J. Funct. Biomater..

[153] Aliotta L., Gigante V., Coltelli M.-B., Cinelli P., Lazzeri C. (2019). Evaluation of Mechanical and Interfacial Properties of Bio-Composites Based on Poly(Lactic Acid) with Natural Cellulose Fibers. Int. J. Mol. Sci..

[154] Aliotta L., Lazzeri C. (2020). A proposal to modify the Kelly-Tyson equation to calculate the interfacial shear strength (IFSS) of composites with low aspect ratio fibers. Compos. Sci. Technol..

[155] Gigante V., Aliotta L., Phuong V.T., Coltelli M.B., Cinelli P., Lazzeri C. (2017). Effects of waviness on fiber-length distribution and interfacial shear strength of natural fibers reinforced composites. Compos. Sci. Technol..

[156] Seggiani M., Cinelli P., Verstichel S., Puccini M., Vitolo S., Anguillesi I., Lazzeri A. (2015). Development of fibres-reinforced biodegradable composites. Chem. Eng. Trans..

[157] Cinelli P., Seggiani M., Mallegni N., Gigante V., Lazzeri C. (2019). Processability and Degradability of PHA-Based Composites in Terrestrial Environments. Int. J. Mol. Sci..

[158] Greene J. (2012). PLA and PHA Biodegradation in the Marine Enviornment.

[159] Seggiani M., Cinelli P., Balestri E., Mallegni N., Stefanelli E., Rossi A., Lardicci C., Lazzeri C. (2018). Novel Sustainable Composites Based on Poly(hydroxybutyrate-co-hydroxyvalerate) and Seagrass Beach-CAST Fibers: Performance and Degradability in Marine Environments. Materials.

[160] Chiellini E., Cinelli P., Imam S.H., Mao L. (2001). Composite films based on biorelated agro-industrial waste and poly(vinyl alcohol). Preparation and mechanical properties characterization. Biomacromolecules.

[161] Chiellini E., Cinelli P., Chiellini F., Imam S.H. (2004). Environmentally Degradable Bio-Based Polymeric Blends and Composites. Macromol. Biosci..

[162] Righetti M.C., Cinelli P., Mallegni N., Massa C.A., Aliotta L., Lazzeri C. (2019). Thermal, Mechanical, Viscoelastic and Morphological Properties of Poly(lactic acid) based Biocomposites with Potato Pulp Powder Treated with Waxes. Materials.

[163] Sakai T. (2013). Screw extrusion technology—Past, present, and future. Polimery.

[164] Gigante V. (2020). Processing and Characterization of PLA/PBAT Blends for Toughened Rigid and Flexible Items. Ph.D. Thesis.

[165] Dhanasekharan K.M., Kokini J. (2003). Design and scaling of wheat dough extrusion by numerical simulation of flow and heat transfer. J. Food Eng..

[166] Shah A., Gupta M. (2004). Proceedings of the Comparision of the Flow in Co-Rotating and Counter-Rotating Twin-Screw Extruders, Annual Technical Conference—ANTEC.

[167] Lazzeri A., Phuong T.V., Cinelli P. (2016). Copolymers Based on Reactive Polyesters and Plasticisers for the Manufacture of Transparent, Biodegradable Packaging Film; 2013.

[168] Mallegni N., Phuong T.V., Coltelli M.-B., Cinelli P., Lazzeri C. (2018). Poly(lactic acid) (PLA) Based Tear Resistant and Biodegradable Flexible Films by Blown Film Extrusion. Materilas.

[169] Cinelli P., Coltelli M.-B., Lazzeri A., Morganti P. (2018). Naturally Made Hard Containers for Food Packaging: Actual and Future Perspectives. Bionanotechnology to Save the Environment; Plant and Fishery’s Biomass as Alternative to Petrol.

[170] Ruys L., van Olmen R. (2012). New Developments in Agrotextiles Based on the Results of the FP7 Project “Bioagrotex”; Grant agreement ID: 213501.

[171] Drumright R.E., Gruber P.R., Henton D.E. (2000). Polylactic Acid Technology. Adv. Mater..

[172] Bonaldi R. (2018). Functional finishes for high-performance apparel. High-Perform. Apparel.

[173] Centexbel—VKC Extrusie (Textiel). https://www.centexbel.be/en.

[174] EU Project EcobioNet. https://ec.europa.eu/environment/eco-innovation/projects/en/projects/ecobionet.

[175] European Commission A European Strategy for Plastics in a Circular Economy. https://ec.europa.eu/environment/circular-economy/pdf/plastics-strategy-brochure.pdf.

[176] Gesellschaft für Verpackungsmarktforschung (2018). Flexible Plastic Packaging Market in Germany and in Europe.

[177] Peelman N., Ragaert P., de Meulenaer B., Adons D., Peeters R., Cardon L., van Impe F., Devlieghere F. (2013). Application of bioplastics for food packaging. Trends Food Sci. Technol..

[178] Jiménez-Rosado M., Zarate-Ramírez L., Romero A., Bengoechea C., Partal P., Guerrero A. (2019). Bioplastics based on wheat gluten processed by extrusion. J. Clean. Prod..

[179] (2019). Global biodegradable plastics market report 2018: Forecast to 2023 featuring NatureWorks, BASF, Total Corbion PLA, Mitsubishi Chemical, and Biome Bioplastics. Focus Catal..

[180] Novamont GmbH. https://germany.novamont.com/page.php?id_page=2&id_first=2.

[181] Piscopo A., Zappia A., de Bruno A., Pozzo S., Limbo S., Piergiovanni L., Poiana M. (2019). Use of biodegradable materials as alternative packaging of typical Calabrian Provola cheese. Food Packag. Shelf Life.

[182] Koide S., Shi J. (2007). Microbial and quality evaluation of green peppers stored in biodegradable film packaging. Food Control..

[183] Khakalo A., Filpponen I., Rojas O.J. (2018). Protein-mediated interfacial adhesion in composites of cellulose nanofibrils and polylactide: Enhanced toughness towards material development. Compos. Sci. Technol..

[184] Reis M.O., Olivato J.B., Bilck A.P., Zanela J., Grossmann M.V.E., Yamashita F. (2018). Biodegradable trays of thermoplastic starch/poly(lactic acid) coated with beeswax. Ind. Crop. Prod..

[185] Soares F.C., Yamashita F., Müller C., Pires A.T. (2013). Thermoplastic starch/poly(lactic acid) sheets coated with cross-linked chitosan. Polym. Test..

[186] Bio-Based News. http://news.bio-based.eu/globally-2014-a-good-year-for-renewables/.

[187] GualapackGroup. https://gualapackgroup.com.

[188] Nawab A., Alam F., Haq M.A., Haider M.S., Lutfi Z., Kamaluddin S., Hasnain A. (2018). Innovative edible packaging from mango kernel starch for the shelf life extension of red chili powder. Int. J. Boil. Macromol..

[189] Nielsen T.D., Holmberg K., Stripple J. (2019). Need a bag? A review of public policies on plastic carrier bags—Where, how and to what effect?. Waste Manag..

[190] Martinho G., Balaia N., Pires A. (2017). The Portuguese plastic carrier bag tax: The effects on consumer’s behavior. Waste Manag..

[191] Lunt J., Bone J. (2001). Properties and dyeability of fibers and fabrics produced from polylactide (PLA) polymers. Aatcc Rev..

[192] Palade L.-I., Lehermeier H.J., Dorgan J.R. (2001). Melt Rheology of Highl-Content Poly(lactic acid). Macromolecules.

[193] Oever M.V.D., Molenveld K., van der Zee M., Bos H. (2017). FBR Sustainable Chemistry & Technology; Bio-based and Biodegradable Plastics: Facts and Figures: Focus on Food Packaging in the Netherlands.

[194] Toray ecodear^®^ Plant-based Synthetic Fibe. https://www.toray.com/products/fibers/fib_0131.html.

[195] Polisilk PLA Filament Yarns. http://www.yarnsource.co.uk/films-fibres-fabrics/.

[196] Swicofil Polyester High Tenacity. https://www.swicofil.com/commerce/products/polyester/165/hightenacity.

[197] Coats Group. https://www.coats.com.

[198] Bio4Pack. https://www.bio4pack.com/de/aktuell/10-jahre-bio4pack-10-jahre-innovation-in-nachhaltiger-verpackung/.

[199] Niaounakis M. (2015). Biopolymers. Processing and Products.

[200] FKuR. https://fkur.com/en/.

[201] Bio-Netting^®^—Netco BV. https://www.netco-bv.nl/en/bio-netting.html.

[202] Hottle T.A., Bilec M.M., Landis A.E. (2013). Sustainability assessments of bio-based polymers. Polym. Degrad. Stab..

[203] Khalil H.A., Davoudpour Y., Saurabh C., Hossain S., Adnan A.S., Dungani R., Paridah M.T., Sarker Z.I., Fazita M.N., Syakir M. (2016). A review on nanocellulosic fibres as new material for sustainable packaging: Process and applications. Renew. Sustain. Energy Rev..

[204] Leceta I., Etxabide A., Cabezudo S., de la Caba K., Guerrero P. (2014). Bio-based films prepared with by-products and wastes: Environmental assessment. J. Clean. Prod..

[205] Detzel A., Kauertz B., Derreza-Greeven C. (2012). Study of the Environmental Impacts of Packagings Made of Biodegradable Plastics. https://www.umweltbundesamt.de/sites/default/files/medien/461/publikationen/4446.pdf.

[206] Vink E.T., Glassner D., Kolstad J.J., Wooley R.J., O’Connor R.P. (2007). Original Research: The eco-profiles for current and near-future NatureWorks^®^ polylactide (PLA) production. Ind. Biotechnol..

[207] Vink E.T., Davies S. (2015). Life Cycle Inventory, and Impact Assessment Data for 2014 Ingeo^™^ Polylactide Production. Ind. Biotechnol..

[208] Hann S., Scholes R., Molteno S., Hilton M., Favoino E., Jakobsen L.G. (2020). Relevance of Biodegradable and Compostable Consumer Plastic Products and Packaging in a Circular Economy.

[209] European Commission (2018). Environmental Impact Assessments of Innovative Bio-Based Products—Summary of methodology and conclusions. Task 1 of “Study on Support to R&I Policy in the Area of Bio-based Products and Services”.

[210] Pilz H., Brandt B., Fehringer R. (2010). The Impact of Plastics on Life Cycle Energy Consumption and Greenhouse Gas Emissions in Europe. https://www.plasticseurope.org/application/files/9015/1310/4686/september-2010-the-impact-of-plastic.pdf.

[211] European Bioplastics Environmental Benefits of Bioplastics. https://www.european-bioplastics.org/.

[212] European Bioplastics Environmental Benefits of Bioplastics.

[213] Plastics Europe The European Association of Plastics Recycling Recovery Organisations. Plastics—The Facts 2017: An analysis of European plastics production, demand, and waste data. https://www.plasticseurope.org/application/files/5715/1717/4180/Plastics_the_facts_2017_FINAL_for_website_one_page.pdf.

[214] Beckham G.T., Johnson C.W., Donohoe B.S., Rorrer N., Mcgeehan J.E., Austin H.P., Allen M.D. (2019). Enzymes for Polymer Degradation.

[215] Narancic T., Verstichel S., Chaganti S.R., Morales-Gamez L., Kenny S.T., de Wilde B., Padamati R.B., O’Connor K.E. (2018). Biodegradable Plastic Blends Create New Possibilities for End-of-Life Management of Plastics but They Are Not a Panacea for Plastic Pollution. Environ. Sci. Technol..

[216] EnviroSim—Wastewater Modeling Software. https://envirosim.com/.

[217] Punch W.F. A System for Predicting the Biodegradability of New Compounds. Research Project Database, Grantee Research Project. EPA Grant Number: R826114. https://cfpub.epa.gov/ncer_abstracts/index.cfm/fuseaction/display.abstractDetail/abstract/949.

[218] Abi-Akl R., Ledieu E., Enke T.N., Cordero O.X., Cohen T. (2019). Physics-based prediction of biopolymer degradation. Soft Matter.

[219] Babu G.L.S., Reddy K.R., Chouskey S.K., Kulkarni H.S. (2010). Prediction of Long-Term Municipal Solid Waste Landfill Settlement Using Constitutive Model. Pr. Period. Hazardous Toxic Radioact. Waste Manag..

[220] EagleVizion—Plastics sorting. http://www.eaglevizion.com/plastics-sorting.

[221] NatureWorks LLC Using Near-Infrared Sorting of Recycle PLA Bottles. https://www.natureworksllc.com/~/media/The_Ingeo_Journey/EndofLife_Options/mech_recycling/20090708_NatureWorks_UsingNIRSortingtoRecyclePLABottles_pdf.pdf.

[222] de Andrade M.F.C., Souza P.M.S., Cavalett O., Morales A.R. (2016). Life Cycle Assessment of Poly(Lactic Acid) (PLA): Comparison Between Chemical Recycling, Mechanical Recycling and Composting. J. Polym. Environ..

[223] Badia J., Ribes-Greus A. (2016). Mechanical recycling of polylactide, upgrading trends and combination of valorization techniques. Eur. Polym. J..

[224] Najafi N., Heuzey M.-C., Carreau P., Wood-Adams P.M. (2012). Control of thermal degradation of polylactide (PLA)-clay nanocomposites using chain extenders. Polym. Degrad. Stab..

[225] Phuong V.T., Gigante V., Aliotta L., Coltelli M.B., Cinelli P., Lazzeri C. (2017). Reactively extruded ecocomposites based on poly(lactic acid)/bisphenol A polycarbonate blends reinforced with regenerated cellulose microfibers. Compos. Sci. Technol..

[226] Takatani M., Ikeda K., Sakamoto K., Okamoto T. (2008). Cellulose esters as compatibilizers in wood/poly(lactic acid) composite. J. Wood Sci..

[227] Coltelli M.-B., Bronco S., Chinea C. (2010). The effect of free radical reactions on structure and properties of poly(lactic acid) (PLA) based blends. Polym. Degrad. Stab..

[228] Coltelli M.-B., Toncelli C., Ciardelli F., Bronco S. (2011). Compatible blends of biorelated polyesters through catalytic transesterification in the melt. Polym. Degrad. Stab..

[229] Henton D., Gruber P., Lunt J., Randall J. (2005). Polylactic Acid Technology. Natur. Fibers Biopolym. Biocom..

[230] Piemonte V., Sabatini S., Gironi F. (2013). Chemical Recycling of PLA: A Great Opportunity Towards the Sustainable Development?. J. Polym. Environ..

